# Proline Metabolism is Essential for *Trypanosoma brucei brucei* Survival in the Tsetse Vector

**DOI:** 10.1371/journal.ppat.1006158

**Published:** 2017-01-23

**Authors:** Brian S. Mantilla, Letícia Marchese, Aitor Casas-Sánchez, Naomi A. Dyer, Nicholas Ejeh, Marc Biran, Frédéric Bringaud, Michael J. Lehane, Alvaro Acosta-Serrano, Ariel M. Silber

**Affiliations:** 1 Laboratory of Biochemistry of Tryps - LaBTryps, Department of Parasitology, Institute of Biomedical Sciences, University of São Paulo, São Paulo, Brazil; 2 Department of Parasitology, Liverpool School of Tropical Medicine, Liverpool, United Kingdom; 3 Centre de Résonance Magnétique des Systemes Biologiques, Université Bordeaux, Bordeaux, France; 4 Department of Vector Biology, Liverpool School of Tropical Medicine, Liverpool, United Kingdom; University of Dundee, UNITED KINGDOM

## Abstract

Adaptation to different nutritional environments is essential for life cycle completion by all *Trypanosoma brucei* sub-species. In the tsetse fly vector, L-proline is among the most abundant amino acids and is mainly used by the fly for lactation and to fuel flight muscle. The procyclic (insect) stage of *T*. *b*. *brucei* uses L-proline as its main carbon source, relying on an efficient catabolic pathway to convert it to glutamate, and then to succinate, acetate and alanine as the main secreted end products. Here we investigated the essentiality of an undisrupted proline catabolic pathway in *T*. *b*. *brucei* by studying mitochondrial Δ^1^-pyrroline-5-carboxylate dehydrogenase (TbP5CDH), which catalyzes the irreversible conversion of gamma-glutamate semialdehyde (γGS) into L-glutamate and NADH. In addition, we provided evidence for the absence of a functional proline biosynthetic pathway. TbP5CDH expression is developmentally regulated in the insect stages of the parasite, but absent in bloodstream forms grown *in vitro*. RNAi down-regulation of *TbP5CDH* severely affected the growth of procyclic trypanosomes *in vitro* in the absence of glucose, and altered the metabolic flux when proline was the sole carbon source. Furthermore, *TbP5CDH* knocked-down cells exhibited alterations in the mitochondrial inner membrane potential (ΔΨ_m_), respiratory control ratio and ATP production. Also, changes in the proline-glutamate oxidative capacity slightly affected the surface expression of the major surface glycoprotein EP-procyclin. In the tsetse, *TbP5CDH* knocked-down cells were impaired and thus unable to colonize the fly’s midgut, probably due to the lack of glucose between bloodmeals. Altogether, our data show that the regulated expression of the proline metabolism pathway in *T*. *b*. *brucei* allows this parasite to adapt to the nutritional environment of the tsetse midgut.

## Author Summary

Bloodsucking insects play a major role in the transmission of pathogens that cause major tropical diseases. Their capacity to transmit these diseases is directly associated with the availability and turnover of energy sources. Proline is the main readily-mobilizable fuel of the tsetse fly, which is the vector of sub-species of *Trypanosoma brucei* parasites that cause human sleeping sickness and are partly responsible for animal trypanosomiasis (*Nagana* disease) in sub-Saharan Africa. Once trypanosomes are ingested from an infected host by the tsetse, the parasites encounter an environment that is poor in glucose (as it is rapidly metabolized by the fly) but rich in proline, which then becomes the main carbon source once the parasite differentiates into the first insect (procyclic) stage. In this work, we provide evidence on the essentiality of *T*. *b*. *brucei* proline catabolism for procyclic survival within the tsetse’s digestive tract, as this organism is unable to synthesize this amino acid and strictly depends on the proline provided by the fly. We also show that parasites deficient in TbP5CDH, a mitochondrial enzyme involved in the proline degradative pathway, failed to proliferate *in vitro*, showed a diminished respiratory capacity, and showed compromised maintenance of energy levels and metabolic flux when proline was offered as the main carbon source. Thus, the integrity of the trypanosome proline degradation pathway is needed to maintain essential functions related to parasite bioenergetics, replication and infectivity within the insect host. Our observations answer a long-standing question on the role of parasite proline metabolism in tsetse-trypanosome interplay.

## Introduction

The study of the metabolic interactions between parasites and insect vectors is critical to understanding their biology and evolution, as well as to aid the design of control strategies that aim to prevent transmission of vector-borne pathogens. Parasites of the *Trypanosoma brucei* sub-species cause sleeping sickness and *Nagana* disease in sub-Saharan Africa, and are exclusively transmitted by tsetse (*Glossina* spp.) flies [1–3]. When *T*. *b*. *brucei* bloodstream forms (BSF) are ingested by a fly, the replicative ‘slender’ trypanosomes rapidly die within the insect midgut (MG), whereas the pre-adapted ‘stumpy’ trypanosomes differentiate into the procyclic form (PF) within 24h [4]. Establishment of a trypanosome infection in the tsetse MG involves parasite colonization of the ectoperitrophic space (a cavity between the peritrophic matrix and the gut epithelium) and subsequent migration to the proventriculus (PV) [5], where the parasite is confined and further differentiates [6]. After multiple morphological and biochemical changes (reviewed in [7, 8]), the parasites then migrate to the salivary glands (SG), where they remain attached to the epithelial cells as epimastigotes ([9] and reviewed in [7]). After colonizing the SG, epimastigotes differentiate into infectious metacyclic forms, which are then released into the fly’s saliva and transmitted to another vertebrate host during a subsequent feed [4].

Unlike most Dipterans, tsetse flies do not store carbohydrates for ATP production [10]. Furthermore, glucose does not seem to constitute a relevant source of energy, is rapidly metabolized (~1h) after the bloodmeal is ingested, and is also found in low amounts in the fluids of these insects [11]. The use of minute amounts of glucose seems to be restricted to the production of other metabolites, such as non-essential amino acids in anabolism-requiring situations, *e*.*g*. pregnancy [12]. Thus, tsetse flies are adapted to efficiently metabolize amino acids and, more specifically, to catabolize proline to accomplish ATP biosynthesis [13, 14], a characteristic that is associated to obligatory blood feeding dipterans [15]. Additionally, proline is important in lactation, it is the metabolite that energetically supports the flight process and it is preferentially utilized by sarcomeres (flight muscle cells), yielding alanine as the main product. In this context, proline is a critical metabolite for tsetse biology [16].

Amino acid metabolism requires a robust transamination network that allows the transfer of amino groups (-NH_2_) to different acceptors, mainly ketoacids. In the specific case of glutamate, -NH_2_ is preferentially transferred to pyruvate, and yields alanine and oxoglutarate, which are the main intermediate products of proline catabolism. In tsetse flies, alanine is produced from proline by muscle cells and is further delivered into the hemolymph, which is then taken up into the fat body cells, for proline production [17]. This newly synthesized proline is, in turn, delivered to the hemolymph and taken up by flight muscle cells [13, 18]. This cycle allows the continuous supply of proline to flight muscles by keeping high proline levels in the hemolymph, which fuels insect flight [19].

During the *T*. *b*. *brucei* life cycle, the parasite goes through a deep metabolic reprogramming; this process allows the parasite to optimize its nutritional requirements according to the available metabolic resources in each environment. This is the case when trypanosomes transit from glucose-rich environment (in the bloodstream of the mammal) to one rich in amino acids (tsetse midgut), which requires a profound metabolic switch (reviewed in [4, 20]). Among the amino acids catabolized, L-proline plays a major role in the bioenergetics of trypanosomes [21–24]. In particular, the procyclic stage of *T*. *b*. *brucei* uses L-proline as a major carbon and energy source [23], which is actively taken up [25] and catabolized inside the mitochondrion into succinate, alanine and acetate with production of intermediate metabolites, reduced cofactors and ATP [26, 27]. Conversion of proline into glutamate is mediated by two enzymatic steps and one non-enzymatic step. First, proline is oxidized into Δ^1^-pyrroline-5-carboxylate (P5C) by a FAD-dependent proline dehydrogenase (TbProDH) [EC 1.5.99.8] [23]. Second, the cyclic P5C ring is spontaneously opened through a non-enzymatic reaction to produce glutamate-γ-semialdehyde (γGS). Third, the carbonyl moiety of γGS is further oxidized to glutamic acid by a P5C dehydrogenase (TbP5CDH) [EC 1.5.1.12] with a concomitant reduction of NAD(P)^+^ into NAD(P)H [28]. Unlike *Trypanosoma cruzi*, there are no genomic or biochemical data supporting the existence of a proline biosynthetic pathway in *T*. *b*. *brucei* [29], which suggests it is auxotrophic for this amino acid. Moreover, in PFs it was reported that proline degradation is downregulated in the presence of glucose [24], and the importance of Ca^2+^ regulation of TbProDH activity in the energy metabolism of trypanosome insect stages was recently suggested [30]. Collectively, both proline oxidation to glutamate and further oxidation through a part of the tricarboxylic acid cycle (TCA) are able to produce reduced equivalents, as well as fuel oxidative phosphorylation, and thus contribute to fulfilling the parasite’s energy requirements [31].

The relevance of proline metabolism for both *T*. *b*. *brucei* and the tsetse led us to address the long-standing question on the role of this amino acid in the parasite´s ability to infect flies. While the importance of TbProDH to the parasite’s biology has previously been studied, little is known on the specific role of TbP5CDH, besides its participation in the complete oxidation of proline. In this work we addressed this issue by studying the role of TbP5CDH in the bioenergetics of *T*. *b*. *brucei* as well as its importance during a tsetse infection. Our data show that in the absence of glucose, *T*. *b*. *brucei* PFs rely on the proline provided by the fly and on a fully functional proline catabolic pathway to successfully survive within the tsetse midgut.

## Results

### TbP5CDH is developmentally regulated among *T*. *b*. *brucei* stages

**Fig 1:**
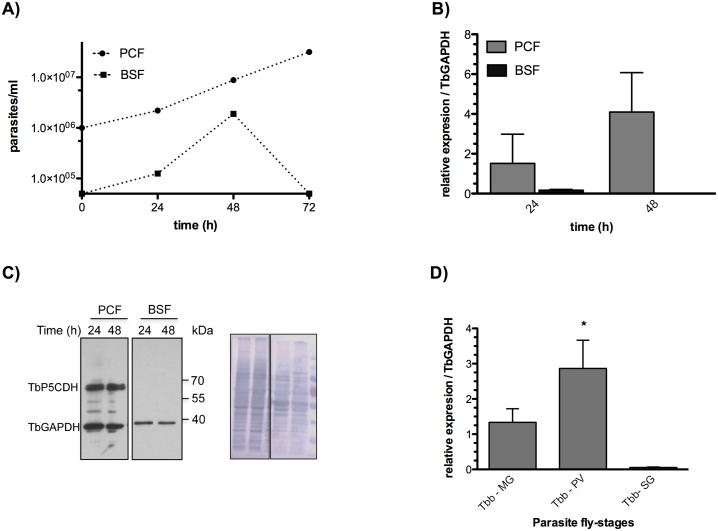
Analysis of TbP5CDH expression levels during the main life stages of *T*. *b*. *brucei*. A) Cell densities from both PCFs and BSFs of *T*. *b*. *brucei* were monitored during 72h of growth. Cell samples were taken at 24h and 48h, and both total-RNA and protein samples were prepared for TbP5CDH expression analysis. B) mRNA expression levels of the *TbP5CDH* were relative to the expression of *TbGAPDH*, as housekeeping gene. Bars represent mean +SD from three biological replicates (n = 3). C) Protein levels were analyzed by western blotting using anti-TcP5CDH (1:2,500) and anti-TcGAPDH (1:4,000) diluted in PBS-T plus 5% (w/v) skimmed milk. Protein relative molecular masses were 63 and 39 kDa for TbP5CDH and TbGAPDH, respectively. Protein loading controls were verified by nigrosine staining of the PVDF membrane after probing with specific antibodies. D) The mRNA levels were determined by qPCR using total RNA of *T*. *brucei*-infected fly tissues (i). Parasites were isolated from the midgut (MG), proventriculus (PV) and salivary glands (SG). Comparisons were made individually and differences were analyzed using two-way ANOVA and Tukey’s post-test. The asterisk (*) denotes the significance gene expression value (p<0.05) of PV over SG samples.

In order to understand the role(s) of TbP5CDH in *T*. *b*. *brucei* biology, we first characterized its expression during the *in vitro* growth of both procyclic cultured forms (PCFs) and BSFs. Parasites were cultured in complete SDM79 and HMI9 media, respectively, and their growth followed up for 72h (although the analyses were made at 24 and 48h depending on the different parasite doubling times; Fig 1A). To analyze the expression profile of *TbP5CDH* and its influence on proline metabolism, *TbP5CDH* mRNA and protein levels were determined by qPCR and western blot, respectively. While both the mRNA and protein levels remained almost constant over time in PCFs, no TbP5CDH protein was detected in BSFs (Fig 1B and 1C). This indicates that, at least *in vitro*, expression of this enzyme is tightly regulated between different trypanosome stages. This observation is consistent with previous data showing that proline catabolism seems to be repressed in *T*. *b*. *brucei* BSFs [32]. We then investigated whether TbP5CDH expression is developmentally regulated during tsetse infection by isolating parasites from different infected organs; i.e. MG, PV and SG. *TbP5CDH* mRNA was detected in parasites collected from the PV and MG but not from SG-derived forms (Fig 1D). No significant changes in the expression levels were observed between PV and MG forms, but there was a strong reduction (60-fold change, p<0.05) in mRNA levels in SG forms. Notably, it was not possible to examine TbP5CDH protein expression by western blotting due to strong cross-reactivity with the *Glossina* P5CDH protein. Collectively, these results suggest that both PV and MG trypanosome forms express the proline-oxidizing pathway, which would be necessary to fulfill the energy requirements for cell proliferation, although the enzyme is downregulated as the infection progresses towards the SGs.

### TbP5CDH is localized to the mitochondrion of procyclic trypanosomes

**Fig 2:**
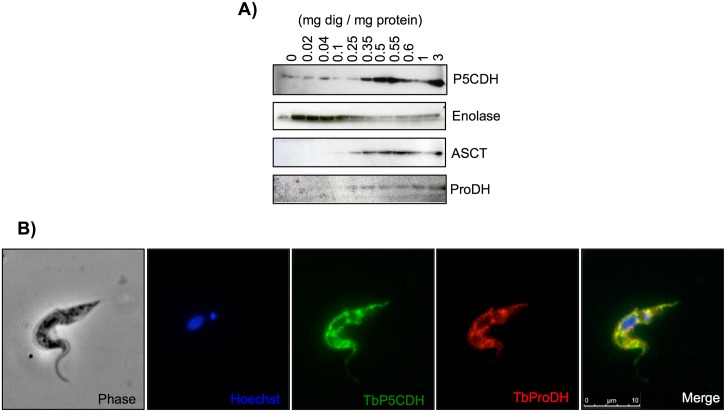
Subcellular localization of TbP5CDH in PCFs. A) Western blot analysis from protein samples obtained after digitonin fractionation. Detection of TbP5CDH in soluble fractions was compared to protein markers. Specific antibodies against trypanosome enolase, acetate:succinyl-CoA transferase (TbASCT) and proline dehydrogenase (TbProDH) were used as cytosolic, mitochondrial (matrix) and mitochondrial-inner membrane markers, respectively. B) Immunolocalization profile of TbP5CDH and TbProDH in PCFs. Cells were visualized under phase contrast. DNA was stained with Hoechst probe (Blue). TbP5CDH (green) and TbProDH (red) were labeled using antibodies produced in mouse and rabbit, respectively.

To determine the subcellular location of TbP5CDH, *T*. *brucei* PCFs were submitted to digitonin fractionation and the enzyme was detected by western blotting. As shown in Fig 2A, TbP5CDH was released together with the mitochondrial markers TbASCT and TbProDH, while the cytosolic marker enolase was released at much lower digitonin concentrations (20 μg compared to 350 μg of digitonin mg^-1^ of protein) [33]. Under these assay conditions, we also detected TbProDH but at low amounts, which is consistent with its possible association with the mitochondrial inner membrane (Fig 2A) [23]. Furthermore, immunofluorescence of fixed PCFs showed co-localization of TbProDH and TbP5CDH (Fig 2B), thus confirming the results obtained by digitonin fractionation.

### TbP5CDH is important for cell growth, production of reducing equivalents and ATP synthesis in the absence of glucose

**Fig 3:**
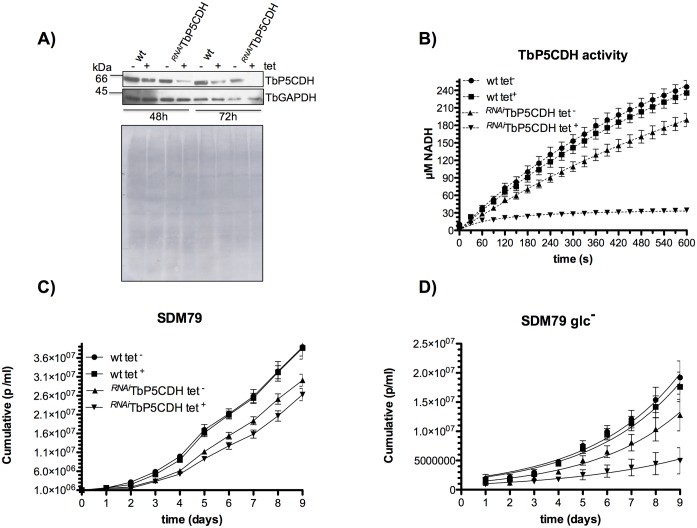
Phenotypic characteristics of *TbP5CDH* RNAi cells. A) Protein levels of TbP5CDH and TbGAPDH after three days of tetracycline-induction. Comparisons were made between non-induced (tet^-^) and tet-induced (tet^+^) from both wt and ^*RNAi*^TbP5CDH mutant cells. Cell lysates (30 μg of total protein per lane) were loaded and probed with antibodies as indicated before. Protein loading controls were verified by nigrosine staining of the PVDF membrane after probing with specific antibodies. B) TbP5CDH activity was determined after three days of tetracycline-induction in wt and RNAi-induced cells. Cell-free total lysates were prepared from PCF trypanosomes and used as enzyme samples. Steady-state rates were monitored spectrophotometrically (Abs_340nm_) using 200 μg of each lysate to start the reaction. C-D) Growth curves of wt tet^-/+^ and ^*RNAi*^TbP5CDH tet^-/+^ PCFs. Parasites (10^6^ cells/ml) were grown in standard SDM79 (C) or SDM79 glc^-^ (glucose-depleted) (D) selective media. Cell densities were determined daily and were split into fresh medium every 72h. Plots represent cumulative cell numbers determined over a period of 9 days.

To determine the importance of TbP5CDH in the bioenergetics of trypanosomes, we downregulated its expression by RNAi using a tetracycline-inducible system [34]. After 72h of tetracycline-induction (^*RNAi*^TbP5CDH tet^+^), no TbP5CDH was detected by western blotting (Fig 3A). However, when we assayed its enzymatic activity, we observed ~16% remaining activity compared to non-induced cells (^*RNAi*^TbP5CDH tet^-^) (Fig 3B). No changes in the levels of TbP5CDH were observed in wt cells supplemented or not with tetracycline (wt tet^-/+^), which showed that addition of this antibiotic had no direct effect on TbP5CDH expression (Fig 3A and 3B).

As previously shown, wt PCFs are able to replicate in standard SDM79 supplemented (or not) with glucose (SDM79 and SDM79 glc^-^, respectively) [23]. In standard SDM79, glucose is the preferred carbon source for PCFs, whereas in the absence of glucose, the parasites mainly use proline as a carbon source and for ATP production. In the case of TbP5CDH, the enzyme was essential when proline was the major carbon source. However, the phenotype was not lethal most likely because of the remaining enzymatic activity in the ^*RNAi*^TbP5CDH cell line (Fig 3C and 3D). These findings prompted us to evaluate the main mitochondrial functions (i.e. ΔΨ_m_, O_2_ consumption rates and ATP levels) in ^*RNAi*^TbP5CDH cells energized with proline.

**Fig 4:**
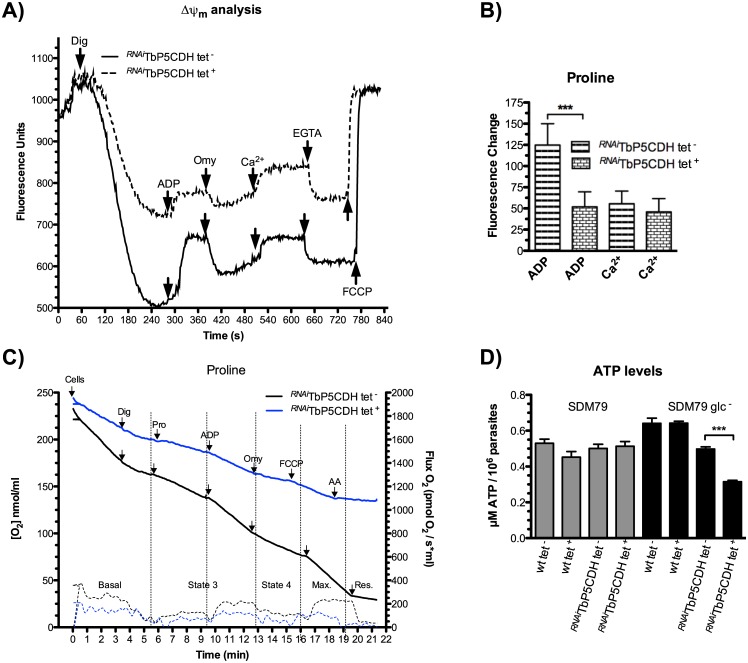
Assessment of mitochondrial function in PCF trypanosomes. The capacity of mitochondrial inner membrane to retain safranin dye was monitored in digitonized cells. Changes in the safranin fluorescence are representative of the mitochondrial inner membrane potential (ΔΨ_m_) in PCFs. ^*RNAi*^TbP5CDH cells were selectively permeabilized with digitonin until fluorescence quenching was stabilized. Then, 250 μM ADP and 50 μM calcium chloride (Ca^2+^) were added to induce depolarization of the mitochondrial (mt)-inner membrane or 0.5 μg/ml oligomycin (Omy) and 500 μM EGTA to revert it, as indicated. Finally, 0.3 μM FCCP was added to collapse the proton gradient, thus releasing the dye. B) Changes in fluorescence obtained after ADP or Ca^2+^ addition were compared between tet^-/+^ cells. Statistical differences were obtained by unpaired t-test (* p<0.05). C) Oxygen consumption rates in PCFs were determined in intact cells (Basal), followed by digitonin addition to selectively permeabilize mt-inner membrane. Then, L-proline (5 mM) was added as mitochondrial substrate and respiration at state 3 was obtained after addition of ADP (250 μM). Inhibition of F_o_/F_1_-ATP synthase was induced by oligomycin addition, to induce the non-phosphorylating respiration (state 4). Maximum respiratory capacity (Max.) was analyzed after induction of non-coupled respiration produced by FCCP (0.3 μM). Finally, the residual oxygen consumption (Res) was determined after addition of mitochondrial inhibitor antimycin A (0.5 μM). The plot is representative of four biological replicates, and mean values were detailed in Table 1. D) ATP levels were determined in wt tet^-/+^ and ^*RNAi*^TbP5CDH tet^-/+^ cells grown in either SDM79 or SDM79 glc^-^ media for three days. ATP concentration was extrapolated from the standard curve. Bars represent mean ± SD of total ATP levels relative to parasite number. Statistical differences were determined using unpaired t-test (*** p<0.01).

In digitonin-permeabilized cells, downregulation of *TbP5CDH* caused a diminished capacity to retain the mitochondrial dye safranin and to respond to the addition of ADP compared to non-induced cells. This profile reflects a partial depolarization of mitochondria from ^*RNAi*^TbP5CDH tet^+^ cells when proline is the electron source for the oxidative phosphorylation (OxPHOS) process (Fig 4A). No changes were observed for the same parameters when succinate was used as a mitochondrial substrate (S1A and S1B Fig). In addition, ADP failed to induce the proton flux into the matrix space through the F_o_/F_1_ ATP synthase complex and did not decrease ΔΨ_m_ to the same levels shown by non-induced cells. Moreover, addition of oligomycin, an inhibitor of ATP synthase, also resulted in a slight increase in ΔΨ_m_, and reestablished the resting levels, which were significantly lower than control. This is likely due to the diminished electron flux from proline degradation to the respiratory complexes in ^*RNAi*^TbP5CDH tet^+^ parasites, which seem to be insufficient to sustain physiological levels of OxPHOS (Fig 4A). Interestingly, addition of Ca^2+^ to these mitochondrial preparations did not affect the ΔΨ_m_ of wt and ^*RNAi*^TbP5CDH cells, which suggests that variations in the electrochemical potential using proline are due exclusively to mitochondrial electron transfer chain (mt-ETC) capacity rather than mitochondrial Ca^2+^ influx (Fig 4B). Observations made at the ΔΨ_m_ level are consistent with the diminished ability of the mutant cell line to consume O_2_ when proline and ADP were present at high concentrations (respiratory state 3), and the high respiration rates are limited by respiratory chain activity [35]. Moreover, the maximal oxygen reduction capacity was dramatically affected in the ^*RNAi*^TbP5CDH tet^+^ cells when FCCP (which collapses the mitochondrial membrane potential) was added to the mitochondrial preparations (Fig 4C, Table 1), and the respiratory control ratio significantly decreased to 1.44 ± 0.02 (Table 1). When succinate was used as the respiratory substrate in control and ^*RNAi*^TbP5CDH tet^-/+^ parasites, no differences in ΔΨ_m_ or O_2_ consumption rates were observed (S1A–S1C Fig). The ATP levels in parasites cultivated in either SDM79 and SDM79 glc^-^ media were also determined. As expected, the absence of TbP5CDH did not affect ATP levels when glucose was present (Fig 4D). Conversely, when ATP synthesis relied on proline oxidation (cells grown in SDM79-glc^-^), the capacity of ^*RNAi*^TbP5CDH tet^+^ cells to produce ATP was diminished (Fig 4D).

**Table 1:** Respiratory capacity of ^*RNAi*^TbP5CDH tet^-/+^ cells.

*I*O_2_ (pmol/s*ml)*10^7^ cells^-1^		
mt-metabolic state	^*RNAi*^*TbP5CDH* tet^-^	^*RNAi*^*TbP5CDH* tet^+^
Basal	43.22 ± 7.18	25.22 ± 2.53
Proline	15.98 ± 3.84	10.61 ± 1.09
State 3	29.04 ± 6.62	15.91 ± 2.68
State 4	11.29 ± 3.47	9.75 ± 0.48
Maximal	30.53 ± 7.57	9.25 ± 2.24
ROX	3.59 ± 1.06	2.53 ± 0.88
RCR	1.83 ± 0.09	1.44 ± 0.02

Values represent mean ± SEM of oxygen flux consumption (*I*O_2_) determined at 28°C in 2.1 ml reaction mix under constant stirring relative to 10^7^ PCFs. Basal respiration corresponds to oxygen consumption in intact cells using endogenous substrates. After digitonin permeabilization, mitochondria were energized by adding 5 mM L-proline followed by 250 μM ADP to induce mitochondrial metabolic state 3. Non-phosphorylating resting respiration (state 4) was induced by oligomycin followed by the maximal respiratory capacity when FCCP was added. Residual oxygen consumption (ROX) was determined by adding antimycin A. The respiratory control ratio (RCR) was calculated by dividing state 3 by state 4 respiration rates.

### Detrimental effect of P5C accumulation in procyclic trypanosomes

**Fig 5:**
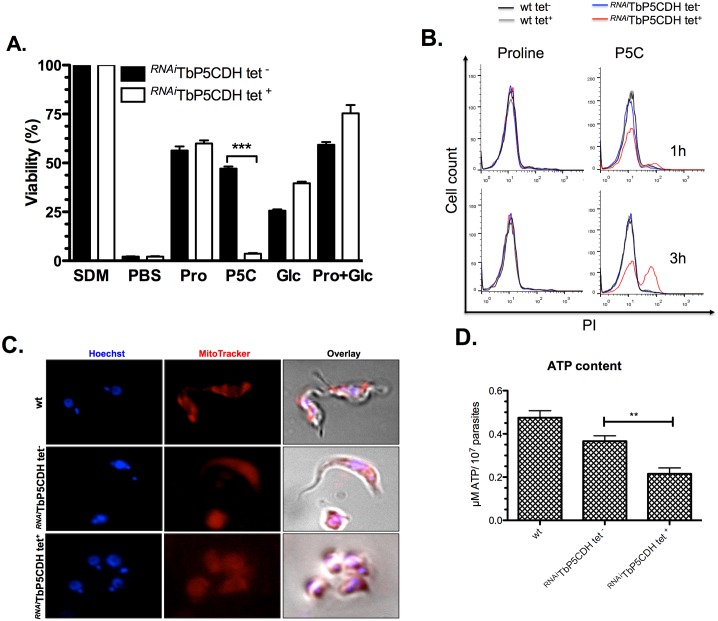
Effect of P5C on cell viability of procyclic forms. A) Cell viability test in PCFs incubated in poor media. ^*RNAi*^TbP5CDH cells were grown for three days in SDM79 and the MTT test performed in the presence of SDM79 (considered as 100% for viability), PBS (positive control), 5 mM proline (pro), 1.5 mM DL-P5C/γGS (P5C), 5 mM glucose (glc) or 5 mM proline plus 5 mM glucose (pro+glc). B) Effect of P5C on membrane integrity of wt and ^*RNAi*^TbP5CDH cells. Control and knocked-down cells were incubated by 3h with PBS added of 5 mM of L-proline (control) or 1.5 mM of DL-P5C. After this time, cells were labeled with 5 μg/ml of PI and analyzed by flow cytometry. C) Fluorescence microscopy of PCFs after 3h of P5C incubation. DNA was labeled with Hoechst probe and MitoTracker was used for mitochondrial staining, as detailed elsewhere. D) ATP content of wt, ^*RNAi*^TbP5CDH tet^-^ and ^*RNAi*^TbP5CDH tet^+^ cells after 3h of P5C incubation.

Given that the *T*. *b*. *brucei* genome does not appear to contain genes that encode putative P5C/γGS metabolizing enzymes (with the exception of TbP5CDH), it is assumed that the proline-glutamate pathway has no branches. On this basis, it is expected that *TbP5CDH*-knocked down cells would produce elevated quantities of intracellular P5C, which has been described as a toxic metabolite in several cell types [36, 37]. Thus, the deleterious effect observed in *TbP5CDH* knockdown cells could be due not only to a diminished efficiency in ATP synthesis but also due to P5C accumulation. To evaluate this, ^*RNAi*^TbP5CDH tet^-/+^ parasites were incubated *in vitro* under different metabolic conditions (i.e. PBS supplemented with L-proline, glucose, proline plus glucose, or P5C/γGS), and their viability was assessed over a 3h period. Controls consisted of ^*RNAi*^TbP5CDH tet^-/+^ cells incubated with either SDM79 (100% viability) or PBS (which yielded a 3% viability compared to cells incubated in SDM79 alone). PCFs incubated in the presence of either proline or proline plus glucose showed a viability of 65% versus SDM79-treated cells, and no significant differences were found for these treatments between induced or not-induced cells (Fig 5A). The addition of P5C/γGS to the ^*RNAi*^TbP5CDH tet^-^ cells resulted in almost the same viability as proline treatment (50%). Notably, incubation of ^*RNAi*^TbP5CDH tet^+^ cells with P5C/γGS reduced their viability by more than 90% (Fig 5A). In addition, non-induced and RNAi-induced procyclics were treated with proline or P5C for 1 or 3h, and P5C toxicity was indicated based on loss of plasma membrane integrity. Only in ^*RNAi*^TbP5CDH tet^+^ cells P5C but not proline treatment resulted in 15% and 57% of Propidium Iodide (PI)-positive cells after 1h and 3h challenge, respectively (Fig 5B). These data were compatible with observed mitochondrial and morphological alterations (Fig 5C). Interestingly, in spite of its deleterious effect, P5C was able to support MitoTracker accumulation (a process that is dependent on the mitochondrial inner membrane potential) and to maintain higher ATP levels in wt or ^*RNAi*^TbP5CDH tet^-^, when compared to ^*RNAi*^TbP5CDH tet^+^ cells. These results, along with previous published evidence [36, 37], suggest that i) P5C is able to reach the mitochondrial matrix; ii) the only metabolic fate for P5C/γGS is to be oxidized to glutamate via TbP5CDH; and iii) the intracellular accumulation of P5C/γGS has a detrimental effect on PCFs viability.

### *T*. *b*. *brucei* PCFs are auxotrophic for proline

**Fig 6:**
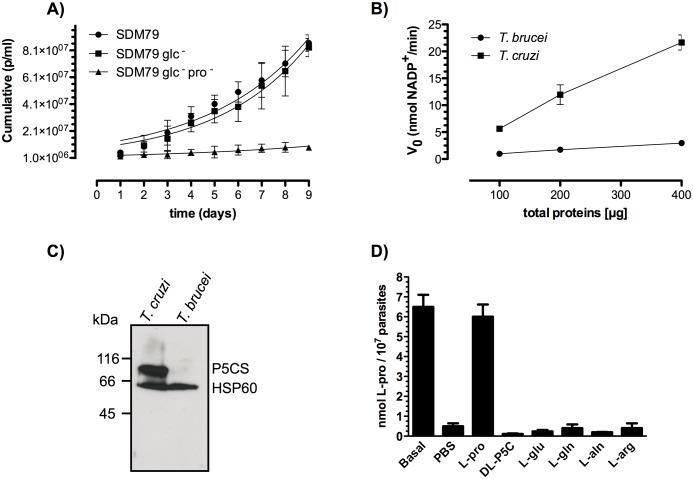
Procyclic forms of *T*. *b*. *brucei* are auxotrophic for proline. A) Growth rates of PCF maintained in complete SDM79, SDM79 glucose-depleted (SDM79 glc^-^) or SDM79 media that contained neither glucose nor proline (SDM79 glc^-^ pro^-^). Cell densities were determined daily, and cells were split every 72h. Plots represent cumulative cell numbers determined over 9 days. B) Enzymatic assay for pyrroline-5-carboxylate reductase (P5CR) activity. Kinetic rates were determined spectrophotometrically by monitoring the NADPH oxidation (Abs_340nm_) resulting from P5C reduction into proline. Activities were measured in total lysates from replicative forms of *T*. *brucei* and *T*. *cruzi* (used as positive control). The plot represents initial velocities (V_0_) in the function of protein variations used in the P5CR assay. C) Detection of pyrroline-5-carboxylate synthase (P5CS) in cell-free lysates. Protein samples from replicative *T*. *b*. *brucei* and *T*. *cruzi* cells (used as positive control) were electrophoresed on SDS-PAGE, blotted onto PVDF membranes and probed with polyclonal antibodies raised against TcP5CS and Heat shock protein (HSP)-60 kDa (TcHSP60), used as reference for protein loads. Expected protein sizes were 81 and 60 kDa for TcP5CS and TcHSP60, respectively. D) Intracellular proline content in PCFs incubated under different precursors. Proline concentration was determined from cells cultivated in SDM79 media (Basal) and after one hour in PBS proline levels were depleted (PBS). Then, proline restoration was assessed over 40 min in the presence of: L-proline (L-pro) used as control, DL-pyrroline-5-carboxylate (DL-P5C/γGS), L-glutamate (L-glu), L-glutamine (L-gln), L-alanine (L-ala), L-arginine (L-arg). Reactants concentrations are detailed in the supplementary data section.

The increased susceptibility of ^*RNAi*^TbP5CDH tet^+^ when exogenous P5C/γGS is added is indicative of the inability of *T*. *brucei* PCFs to reduce it to proline. Thus, we then evaluated whether proline biosynthesis from glutamate or from P5C could happen in *T*. *b*. *brucei*. To address this question, parasites were grown in defined media supplemented or not with proline. When PCFs were grown in either complete SDM79 or SDM79 glc^-^ media no differences were found in the cells doubling time (19.3 ± 1.1 h and 20.3 ± 1.4 h, respectively). After proline deprivation of the media (SDM79 pro^-^ glc^-^), PCFs showed a delay in doubling time (48.4 ± 6h) (Fig 6A). This diminished capability for proliferation under proline-depleted media strongly suggests that *T*. *brucei* is auxotrophic for this amino acid. Furthermore, when the *T*. *b*. *brucei* genome was interrogated for putative genes that encode P5C-synthase (P5CS; converts glutamate into P5C/γGS0) and P5C-reductase (reduces P5C/γGS into proline), using *T*. *cruzi* sequences as queries, only a protein sequence with 65% similarity with *T*. *cruzi* P5CR was found (TritrypDB accession number: Tb927.7.2440). No significant hits were found for P5CS (TritrypDB accession number: TCSYLVIO_005298). The presence of a putative P5CR ortholog in *T*. *b*. *brucei* prompted us to evaluate its enzymatic activity by measuring the reduction of P5C to proline in PCF cell-free extracts. The enzymatic test for P5CR revealed activities of 8.6 ± 0.5 versus 60 ± 9 nmol NADPH/min/mg of protein in *T*. *b*. *brucei* PCF and *T*. *cruzi* epimastigote cell-free extracts, respectively (Fig 6B). Furthermore, P5CS protein was not detected in *T*. *b*. *brucei* lysate using antibodies raised against its *T*. *cruzi* ortholog (Fig 6C). To evaluate the possible occurrence of a proline biosynthetic pathway in *T*. *b*. *brucei* PCFs, the levels of this amino acid were measured in proline–deprived parasites (after 1h incubation in PBS). The cells were then incubated with different substrates that would restore proline levels, i.e. via uptake (proline), reductive biosynthesis (P5C/γGS, glutamate, glutamine), or through the connection between the urea cycle and proline-glutamate pathway (arginine or alanine) as occur in other organisms. Collectively, the demonstration that the only metabolite capable of restoring the normal intracellular levels of proline in PCFs after starvation was proline (Fig 6D) and the lack of genetic and biochemical evidence for a proline biosynthetic pathway in *T*. *b*. *brucei* further corroborate its auxotrophic nature for this amino acid.

### P5C is metabolized by TbP5CDH

As no proline biosynthetic pathway or ornithine transaminase activity could be evidenced in *T*. *b*. *brucei*, TbP5CDH should be the only enzyme capable of metabolizing intra-mitochondrial P5C in these cells. In order to unambiguously evaluate the occurrence of this enzymatic activity we kinetically characterized TbP5CDH from PCF lysates. Our data revealed that these cells were able to reduce NAD^+^ upon the addition of P5C in a concentration dependent manner with apparent K_M_ values of 92.7 ± 14 μM and 0.38 ± 0.04 mM for its substrate (P5C/γGS) and cofactor (NAD^+^), respectively, and V_max_ values of 0.15 ± 0.01 and 0.19 ± 0.01 μmol/min/mg of protein for P5C and NAD^+^, respectively (S2 Fig).

**Fig 7:**
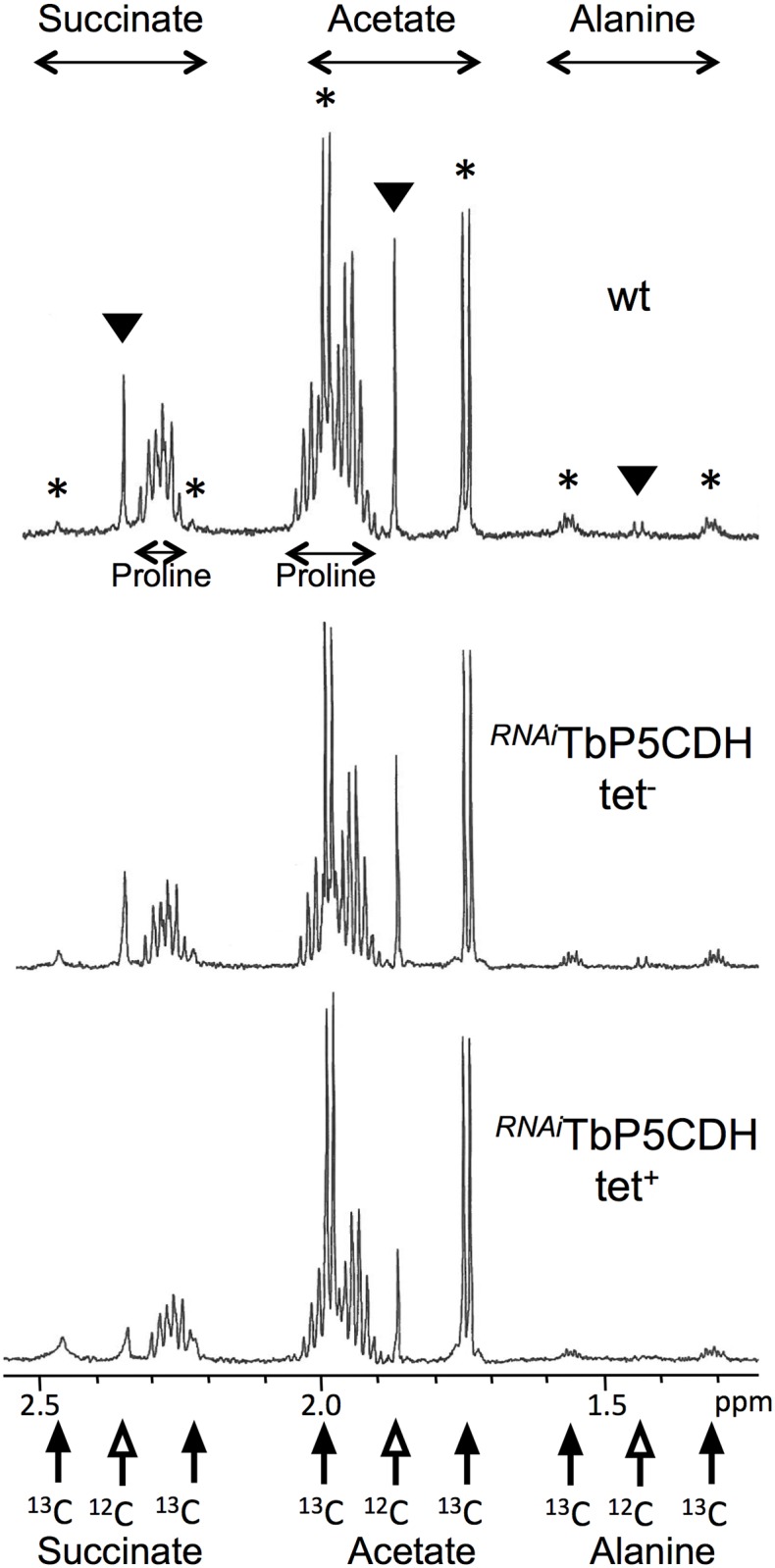
Proton NMR spectroscopy analysis of excreted end products from proline and [U-^13^-C]-glucose metabolism. Metabolic end products (succinate, acetate and alanine) excreted from 4 mM proline and 4 mM [U-^13^C]-glucose by the procyclic wt cell line, as well as the non-induced (tet^-^) and tetracycline-induced (tet^+^) ^*RNAi*^TbP5CDH mutant, were determined by proton NMR spectrometry. Each spectrum corresponds to one representative experiment from a set of five biological replicates. A part of each spectrum ranging from 1.2 ppm to 2.6 ppm is shown. Resonances corresponding to ^13^C-enriched (^13^C) and non-enriched (^12^C) succinate, acetate and alanine molecules are indicated by closed and open arrows below the spectra, respectively, and contribution of proline and [U-^13^C]-glucose to succinate, acetate and alanine is shown in the top panel by arrow heads and asterisks, respectively.

To further determine the metabolic perturbations caused by downregulation of *TbP5CDH*, end products excreted from the catabolism of proline and [U-^13^C]-glucose were analyzed by proton-NMR spectroscopy. We used a previously-developed metabolite profiling assay based on the ability of proton NMR spectroscopy to distinguish ^13^C-enriched from ^12^C molecules [38]. Cells were incubated in PBS with equal amounts (4 mM) of non-enriched proline and of [U-^13^C]-glucose in order to perform a quantitative analysis of proline-derived and glucose-derived acetate production by proton NMR. For instance, [^13^C]-acetate derived from metabolism of [U-^13^C]-glucose (annotated ^13^C in Fig 7) is represented by two doublets, with chemical shifts at around 2.0 ppm and 1.75 ppm, respectively, while the central resonance (1.88 ppm) corresponds to proline-derived [^12^C]-acetate. As expected, the amounts of [U-^13^C]-glucose-derived end products (^13^C-enriched succinate, acetate and alanine) are similar in the ^*RNAi*^TbP5CDH tet^+^ mutant and wt cells (2081 *versus* 2057 nmol excreted/h/mg of proteins), whereas the amounts of excreted end products from proline degradation (non-enriched succinate, acetate and alanine) were 2.2-reduced in the ^*RNAi*^TbP5CDH tet^+^ cell line (Fig 7 and Table 2). The remaining production of end products excreted from proline metabolism (44% compared to wt cells) was probably due to a 16% residual TbP5CDH activity in the tetracycline-induced ^*RNAi*^TbP5CDH mutant. Notably, reduction of succinate and acetate production from proline is compensated by an increased production of these molecules from glycolysis (Fig 7, Table 2). Such flux redistribution towards glucose-derived acetate production was also previously observed in the threonine dehydrogenase procyclic mutant incubated with threonine and [U-^13^C]-glucose [38]. Altogether these metabolic data demonstrate that TbP5CDH is involved in the proline degradation pathway of procyclic trypanosomes.

**Table 2:** Quantitative analysis of excreted end-products from proline metabolism in PCFs.

Metabolic end product	Carbon source	wt tet^- (n = 9)^	^*RNAi*^TbP5CDH tet^- (n = 6)^	^*RNAi*^TbP5CDH tet^+ (n = 5)^
succinate	glucose	89.2 ± 49.5	97.6 ± 25.2	155.4 ± 16.1
	proline	180.8 ± 44.2	173.3 ± 75.6	86.0 ± 13.1
acetate	glucose	1682.6 ± 99.1	1850.7 ± 145.8	1809.2 ± 80.4
	proline	271.2 ± 63.9	216.6 ± 55.2	114.3 ± 28.5
alanine	glucose	285.0 ± 75.5	211.3 ± 86.9	116.8 ± 51.4
	proline	57.5 ± 29	58.2 ± 30.5	27.6 ± 14.4
Total	glucose	2050.9 ± 199.0	2159.5 ± 165.3	2081.4 ± 133.4
	proline	452 ± 160.4	390 ± 110.4	200.3 ± 30.6

Parasites (5x10^8^) were incubated in 5 ml PBS supplemented or not with 5 mM L-proline plus 4 mM [U-^13^C] D-glucose during 6h at 26°C. Cells were harvested by centrifugation and supernatants (~300 μl) were used for NMR analysis. Values represent means ±SD from replicates performed separately (n) and units are expressed in nmol/h/mg protein.

### TbP5CDH is essential for trypanosome survival in the tsetse midgut

**Fig 8:**
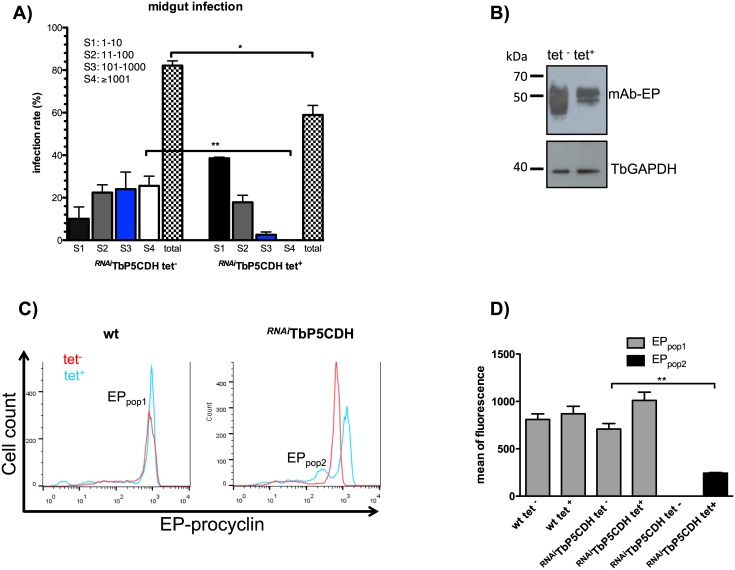
TbP5CDH is essential for establishment of midgut infection and affects procyclin expression. A) Effect of TbP5CDH depletion in PCFs during a midgut colonization assay in tsetse flies. Teneral flies were fed with a bloodmeal that contained 5x10^5^ PCF/ml from ^*RNAi*^TbP5CDH tet^-/+^ cells. Bars represent the percent of trypanosome-infected flies as scored (S1-S4) by microscopy, and the sum of the each scored infection represent the total percent of infected flies per treatment (total). Number of dissected flies (n) for each group were: ^*RNAi*^TbP5CDH tet^-^ (n = 75), ^*RNAi*^TbP5CDH tet^+^ (n = 74). Differences were significant after one-way ANOVA test followed by Bonferroni test (** p<0.001). B) Immunoblotting detection of EP-procyclin in non-induced (tet^-^) and RNAi-induced (tet^+^) parasites grown in SDM79 medium for five days. EP-procyclin was visualized using the anti-EP repeats mAb247 (1:2,000); anti-TcGAPDH (1:4,000) was used as control for protein loading. Expected protein sizes were 39 and ~45 kDa for TbGAPDH and EP-procyclin, respectively. C) FACS analysis of surface expression of EP-procyclin. Comparisons were made in non-induced (tet^-^) and RNAi-induced (tet^+^) cells from wt and ^*RNAi*^TbP5CDH parasites after four days of tet addition. Parasites were fixed (2% (v/v) paraformaldehyde and 0.05% (v/v) glutaraldehyde), incubated with mAb247 (1:500) and then labeled with mouse anti-IgG coupled to AlexaFluor488 (Invitrogen). ^*RNAi*^TbP5CDH tet^+^ group exhibits two different cell populations named as EP-pop_1_ and EP-pop_2_. D) Intensities of mean fluorescence were determined in the cell populations obtained after mAb427 labeling. Values were calculated from four biological replicates (n = 3) and bars represent mean +SD among groups. EP-pop_1_ was observed in both wt and ^*RNAi*^TbP5CDH groups, whereas the EP-pop_2_ population was only displayed in ^*RNAi*^TbP5CDH tet^+^ cells.

After observing differences in the expression levels of TbP5CDH during parasite development in the fly (Fig 1D), we then analyzed its essentiality for parasite survival in the tsetse midgut. Flies were infected with a bloodmeal supplemented with either wt or ^*RNAi*^TbP5CDH PCFs, which were either previously induced or not with tet. At 9 days post-infection (dpi), the flies were dissected and midgut infections were determined. Flies fed with either wt or ^*RNAi*^TbP5CDH tet^-^ cells had infection rates of 82% (Fig 8A, S3 Fig). Furthermore, there were no differences in the number of parasites in the midguts of wt tet^-^, wt tet^+^ or ^*RNAi*^TbP5CDH tet^-^ infected flies (Fig 8A, S3 Fig). However, after downregulation of *TbP5CDH*, the midgut infection rates dropped significantly to 58% (p<0.01) and, importantly, only a few parasites were visible (Fig 8A). Furthermore, under normal TbP5CDH expression (i.e. wt tet^-^, wt tet^+^ or ^*RNAi*^TbP5CDH tet^-^), the infected midguts had a much higher number of parasites (>1000 cells per field) compared to flies infected with ^*RNAi*^TbP5CDH tet^+^ cells (≤10 cells per field) (Fig 8A, S3 Fig). Parasites were probably present in the latter group due to residual expression of TbP5CDH and/or to the transient utilization of glucose present in subsequent bloodmeals. Altogether, these data demonstrate that TbP5CDH activity, a key enzyme in the parasite proline metabolism pathway, is crucial for trypanosome survival within the tsetse fly midgut.

### Alterations in the proline-glutamate pathway affect expression of EP-procyclins

EP- and GPEET-procyclins are the most abundant GPI-anchored surface glycoproteins on the surface of *T*. *b*. *brucei* PCFs. The C-terminus of all EP-isoforms contains abundant (up to 30) repeats of glutamate (E) and proline (P) dipeptides [55]. Likewise, GPEET-procyclin is also rich in E and P because of its 5–6 GPEET C-terminal repeats. We investigated whether alterations in the proline-glutamate oxidative flux interfere with the expression of all procyclin isoforms. Western blotting analysis showed a slightly decreased in EP-procyclin expression in ^*RNAi*^TbP5CDH tet^+^ cells compared to wt tet^-^, wt tet^+^ (Fig 8B). Interestingly, perturbations in the number of EP-positive cells were found after four days of RNAi-induction for TbP5CDH. Two different cell populations were observed, which were named as EP-pop1 and EP-pop2 (Fig 8B, right panel). The EP-pop1 displayed similar values of fluorescence intensity versus controls (wt tet^-/+^ or ^*RNAi*^TbP5CDH tet^-^ (Fig 8C), whereas the EP-pop2 population showed a 10-fold reduction in the mean of fluorescence. However, when the repertoire of procyclins was analyzed by MALDI-TOF (S4A–S4D Fig) mainly EP1-2 and EP3 isoforms (containing 25 and 22 EP repeats, respectively [55]) were detected in either induced or non-induced cells. This suggests that although the overall expression of EP-procyclins appears to be slightly compromised when the proline metabolism pathway is altered (Fig 8C), these cells do not seem to compensate the slight EP deficit by re-expressing GPEET-procyclin.

## Discussion

Once *T*. *b*. *brucei* blood forms are ingested by the tsetse, differentiation of stumpy trypanosomes to procyclics is triggered by a combination of at least two key factors that modulate parasite gene expression [39], i.e. a drastic decrease in temperature and the presence of specific metabolites inside the fly’s gut. Many developmental changes allow parasites to adapt to the midgut’s hostile environment, including the expression of a procyclin coat, which helps to protect the parasite surface against tsetse midgut proteases [40], and development of a functional mitochondrion for energy production. In this work, we biochemically and genetically characterize TbP5CDH, an essential mitochondrial enzyme involved in L-proline catabolism. Trypanosomes deficient in the expression of TbP5CDH failed to proliferate *in vitro* in the absence of glucose (which mimics the tsetse midgut environment) and showed compromised mitochondrial activity. Thus, the integrity of the proline degradation pathway in *T*. *b*. *brucei* is needed to maintain essential functions related to parasite bioenergetics, replication and infectivity within the insect host. We further demonstrated that *T*. *b*. *brucei* is unable to produce L-proline; instead it utilizes the proline available in the tsetse midgut. Collectively, our observations confirm the long-standing suggestion that proline metabolism in *T*. *b*. *brucei* is essential for *in vivo* energy production, thus ensuring the viability of infection within tsetse fly.

### Proline metabolism in parasitized tsetse flies

**Fig 9:**
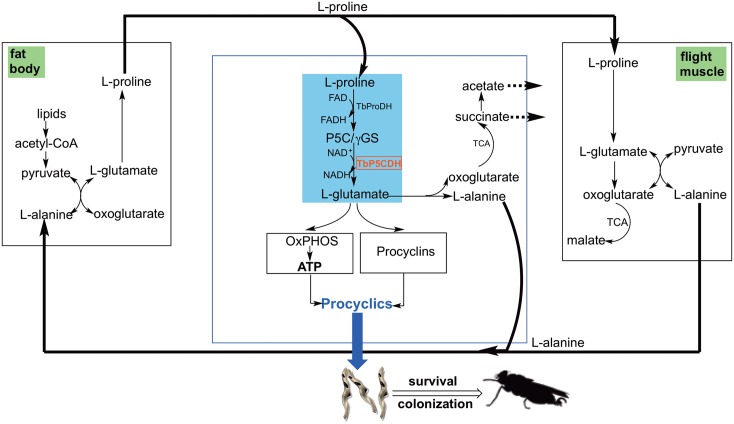
Scheme representing the proline-alanine cycle that occurs between *T*. *b*. *brucei* and both tsetse tissues, fat body and flight muscles. Proline combustion occurs in tsetse flight muscle (right panel), which produces alanine as the main end product. Alanine is transported to the fat body by the hemolymph (left panel). Alanine and lipids constitute the major sources for proline synthesis in the fat body. Thus, in a transamination reaction, the amino group (-NH_2_) is transferred from alanine to oxoglutarate to yield glutamate and pyruvate. Pyruvate can be carboxylated to form oxaloacetate while β-oxidation of lipids becomes the main source of acetyl-CoA. The fat body TCA cycle goes from citrate to oxoglutarate, the latter which can be an acceptor of -NH_2_ in a new transamination reaction to produce glutamate. This glutamate is further reduced to proline, which is then transported by the hemolymph to the flight muscles. In the flight muscles proline is oxidized to glutamate, which acts as a donor of -NH_2_ in a new transamination reaction in which pyruvate is the acceptor, forming alanine and oxoglutarate [15]. Glutamate can also be deaminated to form oxoglutarate through glutamate dehydrogenase activity. Oxoglutarate is decarboxylated and oxidized to malate through the TCA cycle. The malic enzyme converts malate into pyruvate, which can in turn be a new acceptor of -NH_2_ transferred from glutamate (to form alanine and oxoglutarate, as described above) [42]. During a *T*. *b*. *brucei* infection, the parasites use part of the proline produced in the fat body and transported by the hemolymph to proliferate and colonize the MG (blue section). In *T*. *b*. *brucei*, the first steps of the proline catabolic pathway are similar to those of insect muscle cells: proline is oxidized to glutamate, which can either be converted to oxoglutarate after deamination or transaminated to produce alanine and oxoglutarate. Unlike in tsetse, oxoglutarate can be further converted into succinate, which is excreted into the extracellular medium. Succinate can also be converted into malate, which is further decarboxylated to produce pyruvate. An additional decarboxylation of pyruvate yields acetyl-coA, which can be used to produce acetate that is excreted to the extracellular medium. Succinate, acetate and alanine are the major excreted products of *T*. *b*. *brucei* insect forms resulting from proline degradative flux (dotted arrows). Alanine is excreted and could reach the hemolymph during procyclics proliferation, thus enriching the insect pool of available alanine. *T*. *b*. *brucei* also uses glutamate produced from proline for the synthesis of EP-procyclins, which are needed for midgut procyclic development. In addition, proline is critical to fuel electrons to support mt-inner membrane potential, respiratory capacity and ATP synthesis driven by OxPHOS. Midgut procyclics strictly depend on proline degradation capability for survival within tsetse fly midgut and TbP5CDH (in red) is essential for colonization and establishment of parasite infection within tsetse.

Some Dipterans (including the genus *Glossina*) are well adapted to use amino acids for energy production. In fact, due to the scarce carbohydrate reserves in tsetse, glycolytic activity is negligible within this insect [11, 41]. Three characteristics make proline a readily mobilizable energy source in tsetse: i) its highly reduced state, which is related to its high yield in terms of metabolic energy production (i.e. 5-fold more efficient than carbohydrates); ii) its high solubility (allowing its transport in high concentrations, thus permitting an efficient distribution in the entire fly body); and iii) its low nitrogen content limiting the amount of energy required for nitrogen detoxification (reviewed in [42]). Einar Bursell concluded that "proline constitutes the only effective substrate for flight metabolism" for species in which both sexes are obligatory blood-feeders (i.e. *Glossina* spp.) [15]. In fact, proline represents ~4% of the total amino acid content in the tsetse hemolymph and is efficiently burnt during the flight process [43, 44]. It is first oxidized to glutamate, and further converted into oxoglutarate by either an alanine transaminase or a glutamate dehydrogenase (Fig 9, left panel). Consequently, flight time in tsetse (which is limited to about three minutes) is likely to be determined by the amount of proline available in the hemolymph at the outset [43].

On the other hand, the synthesis of proline from alanine in tsetse takes place in the fat body. It is a complex process that comprises an alanine-glyoxylate transaminase, a pyruvate dehydrogenase and part of the TCA cycle. Part of the oxoglutarate produced is converted into glutamate in the same transamination reaction in which a new alanine molecule is converted into pyruvate to feed again the TCA cycle (Fig 9, right panel) [17, 18, 45]. Thus, there is a strong interdependence between proline/alanine metabolism between the fat body and flight muscles of tsetse, which are metabolically connected through the hemolymph. Notably, this metabolic system does not work at the steady state: the release of CO_2_ by the flight muscles creates a deficit of carbon. This deficit is possibly compensated by using Acetyl-CoA from the β-oxidation of lipids in the fat body for proline *de novo* biosynthesis [17].

During a trypanosome infection, parasite colonization of different tsetse organs may alter the fly’s proline-alanine cycle. Such an alteration would not only have an impact on the activity of flight muscles, but also affects tsetse reproduction [45]. The crosstalk in the utilization of proline in trypanosome-infected flies becomes even more complex with the dependency on *Wigglesworthia glossinidia* (the obligate tsetse bacterial symbiont) for the production of vitamin B6, which is essential for activity of alanine-glyoxylate aminotransferase (involved in proline regeneration in fat body) (reviewed in [16]).

### Proline is the major energy and carbon source for midgut forms of *T*. *b*. *brucei*

It has previously been shown that TbProDH is essential when parasites are grown in the presence of proline as the main energy source (SDM79 glc^-^) [23]. In the present work, a different phenotype was observed: TbP5CDH knockdown cells did not die *in vitro*. However, the essentiality of TbP5CDH for survival in the fly was evident by the low midgut infection phenotype in knockdown cells. This discrepancy between the *in vitro* phenotypes could be due to the residual activity (~16%) of TbP5CDH in the tetracycline-induced ^*RNAi*^TbP5CDH mutant, which would be able to maintain a low but significant metabolic flux thus allowing proline oxidation in the TCA cycle/ETC. Alternatively, the activity of TbProDH, a FAD dependent enzyme, might also be able to directly transfer electrons to the ubiquinone pool at the ETC (similarly to succinate dehydrogenase), as already proposed for *T*. *cruzi* [21]. Both possibilities, individually or combined, could explain parasite survival by partially fulfilling the energy requirements of these cells. In *T*. *b*. *brucei*, proline conversion into P5C/γGS produces FADH_2_, which can transfer 2e^-^ to the UQ pool with further reduction of cytochromes [21]. γGS is then converted into glutamate to produce NADH. In addition, four additional reactions downstream to the proline-glutamate conversion produce NADH in *T*. *b*. *brucei* (reviewed in [29]). *T*. *b*. *brucei* expresses a mitochondrial NADH: ubiquinone oxidoreductase (which is rotenone insensitive), uses FMN as cofactor, transfers one e^-^ to the ubiquinone (UQ) pool and can reduce O_2_ to O_2_^-•^ anion [46]. This enzyme is likely to be involved in the reoxidation of NADH, thus reducing UQ and driving proton pumping at level of C-III and C-IV in the mt-ETC [47]. Then, both proline oxidation steps generate reducing equivalents that feed the OxPHOS, thus driving the ATP synthesis through the F_o_F_1_/ATP synthase. The intramitochondrial glutamate produced from proline can either be: i) deaminated into oxoglutarate or ii) transaminated to pyruvate forming alanine. Oxoglutarate can be converted into succinyl-CoA and then into succinate, constituting two points of ATP generation, by substrate level phosphorylation (at succinyl-CoA synthetase level) and OxPHOS (via succinate dehydrogenase complex), respectively [48]. Succinate can also be excreted as an end product. In the absence of glucose, alanine is also excreted by *T*. *b*. *brucei* PCFs as the end product of proline degradation. It may be possible that tsetse also utilizes trypanosome-excreted alanine for further conversion into proline, especially in highly infected tissues.

### Importance of P5C in *T*. *b*. *brucei* proline metabolism

An intriguing phenotype evidenced in our ^*RNAi*^TbP5CDH cell line, was the cell toxicity displayed when exogenous P5C/γGS was added to tet-induced parasites. In most eukaryotes, P5C/γGS can be synthesized by proline oxidation, glutamate reduction or by loss of the -NH_2_ group at the δ-carbon of ornithine through an ornithine transamination reaction. In turn, P5C/γGS can be decreased by its oxidation to glutamate by P5CDH, its reduction to proline, or by its amination to form ornithine [49]. Thus, the amount of free P5C/γGS mainly results from the balance between all these enzymatic activities. *T*. *b*. *brucei* lacks a functional urea cycle, which eliminates any connection between this pathway and P5C/γGS [50]. Furthermore, our results show that neither relevant enzymatic activities related to proline biosynthesis nor genes encoding the putative enzymes for these pathways are present in the *T*. *b*. *brucei* genome. In addition, there was a cytotoxic effect for externally added P5C/γGS to ^*RNAi*^TbP5CDH tet^+^, which supports the oxidation to glutamate as the only fate for this metabolite in PCFs. It should be noted that these results differ to those obtained when *TbP5CDH*-knockdown cells were treated with proline (resulting in the intracellular accumulation of P5C/γGS due to the TbProDH activity), which indicated the cells remained viable (although non-replicative). This was also consistent with the viability shown by cells with increased levels of P5C/γGS by overexpressing a mitochondrial carrier (TbMCP14) [51]. Altogether our results showed that, unlike *T*. *cruzi* –whose energetic metabolism also relies on proline consumption [21, 28]-, *T*. *b*. *brucei* PCF is auxotrophic for proline, this being an essential metabolite and a main carbon source during this stage. As a consequence, P5C/γGS levels depend exclusively on the balance between its formation from proline oxidation and its depletion by oxidation to glutamate. In addition, we confirmed that proline deprivation dramatically affects cell proliferation as previously suggested [52]. Altogether, our data provide evidence that *T*. *b*. *brucei* has a strict requirement of a complete proline to glutamate oxidation pathway to successfully colonize tsetse midguts.

### The impact of proline metabolism on EP-procyclin expression

The relevance of EP-procyclin expression for the successful development of *T*. *b*. *brucei* within the fly has been widely reported [53, 54]. Expression of procyclins (GPEET and EP) varies according to the parasite stages in the tsetse [40]. There seems to be a correlation between the expression of such molecules in midgut forms and elevated mitochondrial activities [55]. More specifically, GPEET expression (normally in early stages) can be reactivated in late forms when mitochondrial activities such as the ASCT cycle or alternative oxidase are inhibited [55]. It was also stated that glycolytic activity, disrupted by RNAi-silencing of the trypanosome hexokinase gene, produces a switch in the surface expression from EP- to GPEET-procyclin [56]. We observed herein that alterations in the proline-glutamate pathway slightly affects the levels of EP-procyclin expressed at the surface. However, this alteration did not induce a change in the type of procyclins these cells expressed and no evidence of GPEET re-expression was observed. Given the specificity of the anti-EP mAb 247 for the glu-pro dipeptides [57], it is likely that the alterations in EP-procyclin expression after down-regulation of *TbP5CDH*, could be simply due to a reduction in the overall levels of intracellular glutamate available for making the C-terminus glu-pro repeats. Furthermore, it is unlikely that such a small reduction in the surface expression of EP-procyclins accounts for the inability of ^*RNAi*^TbP5CDH tet^+^ cells to colonize the tsetse midgut, although EP-procyclin-null trypanosomes are less efficient in establishing midgut infections [53, 54]. Thus, these results further confirm that the fly phenotype observed in knocked down cells appears to be mainly a direct consequence of an interrupted proline metabolism pathway in these parasites.

## Materials and Methods

### Ethics statement

Animal experiments in this work were performed in accordance with the local ethical approval requirements of the Liverpool School of Tropical Medicine and the UK Home Office Animal (Scientific Procedures) Act (1986) under license number 40/2958.

### Trypanosome strains and culture

BSF of *T*. *b*. *brucei* TSW196 strain [58], which is a fully fly-transmissible, was used for gene expression studies and proline determination in infected flies. BSF *T*. *b*. *brucei* from 2T1 strain (kindly provided by David Horn, University of Dundee, UK) were cultured in HMI-9 medium supplemented with 15% (v/v) FCS (Gibco) at 37°C and 5% CO_2_ [59]. The initial cell density was 5x10^4^ cells/ml, which was sub-cultured each 48h. Parasite densities were determined by cell counting using a hemocytometer. PCFs of *T*. *b*. *brucei* Lister 427 (29–13 clone) (*T7-RNAp*^*+*^ *NEO*^*+*^ *TET*^*+*^ *HYG*^*+*^), which expresses the T7 RNA polymerase under the control of the tetracycline (tet) promoter, was cultured *in vitro* in SDM79 media (Gibco) supplemented with GlutaMAX (Gibco), 7.5 μg/ml hemin and 10% (v/v) heat-inactivated fetal calf serum (FCS) [60, 61] at 26°C. For RNAi experiments, parasites were grown in SDM79 media supplemented with 25 μg/ml G418 (G) and 12.5 μg/ml hygromycin (H) as indicated [62]. To grow PCFs in defined medium, we used SDM79 base media without sodium bicarbonate, glucose, glutamine, glutamate, proline, pyruvate, threonine and acetate (SDM79-CGGGPPTA) (PAA laboratories, Pasching, Austria) and then supplemented, except for glucose (SDM79 glc^-^) or proline (SDM79 glc^-^ pro^-^) as indicated [23]. In both cases, the preparation was supplemented with 10% (v/v) tet-free FCS (Clontech Laboratories) and an excess of 50 mM *N*-acetyl-D-glucosamine (GlcNAc) to inhibit uptake of glucose presented in serum (about 1.5 mM) [63]. The initial cell density was 10^6^ cells/ml and sub-culturing was done every 72h [60].

### Tsetse flies

*Glossina morsitans morsitans* flies were maintained in a laboratory colony at the Liverpool School of Tropical Medicine (LSTM) at 26°C and 65–70% relative humidity. Teneral (12-24h post-emergence) flies were fed on sterile defibrinated horse blood (TCS Biosciences Ltd., Buckingham, UK).

### *TbP5CDH* expression

Fly-derived trypanosomes were isolated from the MG, PV and SG of infected flies, as described. Parasites were resuspended in SDM79 medium and midgut debris were removed by filtration through cytometer-tubes filter (Becton Dickinson). Cells were harvested by centrifugation (2,000 *g* for 10 min at 4°C), washed twice with cold PBS (137 mM NaCl, 2.7 mM KCl, 8 mM Na_2_HPO_4_, 1.5 mM KH_2_PO_4_ adjusted to pH 7.3), counted and stored at -80°C until RNA or protein analysis. Total RNA extractions from fly-derived parasites (~5x10^6^ cells) were performed with TriZol reagent (Sigma) following standard procedures [64]. Then, 300 ng total-RNA were used for cDNA synthesis with oligo (dT)_20_ and *SuperScript III Reverse Transcriptase* (RT) (Invitrogen). Resultant cDNA samples were diluted (1:4) in nuclease-free water (NFW) for use in quantitative RT-PCR (qPCR). Based on DNA-sequences for *TbGAPDH* and *TbP5CDH* (TritrypDB accession numbers: Tb927.6.4300 and Tb427.10.3210, respectively), specific primers were designed (S1 File for oligonucleotides sequences). qPCR reactions were performed in 96-wells (Stratagene, Agilent Technologies, La Jolla, TX, USA) using 3.2 pg of each primer, 5 μl fast SYBR green master mix (Applied Biosystems, Life Technologies, CA) and 5 μl of cDNA samples to a final volume of 20 μl per well. Reactions were run in a Mx3000P qPCR-system (Stratagene) followed by a dissociation curve. Samples from naïve tissues were also used to verify primer specificity.

### RNAi of *TbP5CDH*

The pZJM vector, which contains a cloning site between two opposing T7 promoters, was used to silence *TbP5CDH* expression (Tb427.10.3210) [34]. A 5ʹ DNA fragment (480 bp) corresponding to *TbP5CDH* was amplified by conventional PCR using specific primers (see S1 File for oligonucleotides sequences), cloned into the pZJM vector (pZJM/^*RNAi*^TbP5CDH) and the resulting construct was confirmed by sequencing. Plasmid preparation was done using the QIAGEN plasmid Maxi Kit according to the manufacturer’s instructions (QIAGEN). For transfections, 10 μg pZJM/^*RNAi*^TbP5CDH was linearized by digestion with the restriction endonuclease *Not*I (Thermo Scientific), precipitated by standard procedures and dissolved in NFW. PCF trypanosomes (2x10^7^ cells maintained in mid-log phase in SDM79 H/G medium) were transformed using a Nucleofector transfection system II/2b, following the manufacturer’s instructions (Lonza). Parasites were seeded into 24-well plates (<10 cells/well) and cloned by limiting dilution in SDM79 H/G supplemented with 2.5 μg/ml phleomycin as a selection marker. The obtained parasite lineages were referred to as wt (parental Lister 427 29–13 strain) or ^*RNAi*^TbP5CDH, as the RNAi-TbP5CDH cell line, in the presence or absence of tetracycline (tet^-/+^). RNAi was induced by adding 0.5 μg/ml tetracycline disodium salt (freshly dissolved in PBS) to the selective media (at 26°C).

### Infection phenotype analysis of *TbP5CDH*-knockdown cells

Teneral flies were infected with bloodmeal preparations that contained either wt or ^*RNAi*^TbP5CDH parasites. Briefly, non-induced (tet^-^) or tetracycline-added (tet^+^) parasites were added to sterile horse blood at a density of 5x10^5^ parasites/ml. RNAi induction was maintained by adding 25 μg/ml tetracycline to the bloodmeal, and 24h after receiving an infectious blood meal, the flies were sorted and only fed flies were used. After nine days, flies were dissected and the number and intensity of infected midguts was determined by microscopy. A score was attributed to each infection as previously described [65].

### Enzymatic assays

Enzymatic determinations for both P5C reduction to proline or P5C oxidation to glutamate were performed. The substrate of TbP5CDH, a racemic mixture of DL-Δ^1^-pyrroline-5-carboxylate (DL-P5C) and its ring-open form gamma-glutamate semialdehyde (γGS), was synthesized from peroxidation with NaIO_4_ (Sigma), and eluted in acidic medium (1 M HCl) as previously described [66]. The steady-state activity for TbP5CDH was measured in cell-free homogenates from PCFs, as previously described for *T*. *cruzi* [28]. The TbP5CDH reaction mixture contained: 0.3 mM P5C/γGS (freshly prepared), 1 mM nicotinamide adenine nucleotide disodium salt (NAD^+^) and 90 mM potassium phosphate buffer pH 7.2, made up to a final volume of 3 ml with distilled water. The reaction was started after adding 200 μg cell-free homogenates from PCFs and the linear rate was determined by following the increase in absorbance (λ_340nm_) over 5 mins at 28°C with constant stirring. A blank without substrate (P5C/γGS) was used as a control. Readings of samples and controls were made in parallel in a double-beam Thermo *Evolution 300* spectrophotometer (Thermo Scientific). Kinetic parameters for P5C and the cofactor of TbP5CDH were also determined in PCF homogenates. Substrate dependence was assayed by varying the P5C/γGS concentrations over the range of 20–600 μM (freshly prepared) and 1 mM of NAD^+^ as saturating concentration. Cofactor dependence was assayed by varying the NAD^+^ concentrations over the range of 0.01–2.5 mM and 600 μM P5C/γGS as saturating concentration. The P5C-reductase reaction mixture contained: 500 μM P5C/γGS (freshly prepared), 50 μM NADPH and 100 mM Tris-HCl pH 7.0, and was made up to a final volume of 1 ml with distilled water. The reaction was started by adding different concentrations of PCF homogenates. The linear rate was determined by following the decrease in absorbance (λ_340nm_) over 3 min at 28°C with constant stirring. P5C-reductase enzymatic activity determinations from *T*. *cruzi* homogenates were used as controls under the same conditions.

### Determination of free proline in procyclic trypanosomes

Parasites (Lister 427 29–13 strain) were incubated in PBS (for 1h) to diminish the intracellular pool of free proline. Parasites were then incubated for 40 min in the presence of different carbon sources and cofactors (S2 File for detailed mix composition) to determine which combination was able to restore the intracellular proline levels. Additional treatments consisted of parasite incubation with PBS supplemented with 5 mM L-proline (positive control) or non-supplemented PBS (negative control). Parasites were then washed with cold PBS and centrifuged (3,000 *g* for 5 min at 4°C). Pellets were resuspended in 100 μl lysis buffer (100 mM Tris-HCl pH 8.1, 0.25 M sorbitol, 1 mM EDTA, 1% (v/v) Triton X-100, 1 mM phenylmethanesulfonylfluoride (PMSF), 4 μg/ml aprotinin, 10 μg/ml tosyl-L-lysyl-chloromethane hydrochloride (TLCK) and 10 μM E-64), and submitted to two cycles of snap freezing in liquid nitrogen thawing. Crude extracts were clarified by centrifugation (15,000 *g* for 15 min at 4°C) and 100 μl supernatant was mixed (in a separate reaction) with 1 volume 20% (w/v) trichloroacetic acid for deproteinization. Samples were precipitated by centrifugation (20,000 *g* for 30 min at 4°C) and 200 μl of the resultant supernatants were used for the Bates assay, as described elsewhere [67].

### Viability of TbP5CDH cells under different metabolic conditions

Non-induced and RNAi-induced (tet^-/+^) PCFs from wt and ^*RNAi*^TbP5CDH cell lines were grown for three days in SDM79 media at 26°C. Then, parasites were harvested by centrifugation and resuspended in either PBS or PBS supplemented with 5 mM L-proline, 1.5 mM P5C/γGS and 5 mM D-glucose, or with 5 mM L-proline + 5 mM D-glucose, and further incubated for 4h at 26°C. Cell viability was evaluated after incubation with 3-(4,5- dimethylthiazol-2-yl)-2,5-diphenyltetrazolium bromide (MTT) [68]. Results were obtained from three biological replicates (n = 3). Comparisons between non-induced and RNAi-induced cells were calculated using the one-way ANOVA test in GraphPad Prism v5.0a for Mac OS X (GraphPad Software, USA).

### Digitonin titration

PCFs (Lister 427 strain, 29–13 clone) cultivated at the late logarithmic phase of growth (3x10^9^ total cells) in SDM79 medium were harvested by centrifugation [1,000 *g* for 10 min at room temperature (RT°)] and washed twice with PBS buffer. Total protein concentration was determined by the Bradford method [69] and the final pellet was resuspended in STEN buffer (250 mM sucrose, 25 mM Tris-HCl pH 7.4, 1 mM EDTA, 150 mM NaCl, 1 mM DTT and sigma-protease inhibitor mixture) adjusted to a final concentration of 1 mg/protein in 200 μl. Cells were treated with variable concentrations of digitonin (dissolved in STEN + dimethylformamide 40 mg/ml) in a final volume of 300 μl for each treatment, incubated for 4 min at 25°C and centrifuged (2 min at max speed), as previously described [70]. Supernatants corresponding to solubilized fractions were mixed with 1x SDS Laemmli buffer and analyzed by western blotting.

### Western blotting

The presence of TbP5CDH, TbP5C-synthase, acetate:succinyl-CoA transferase (ASCT), enolase, TbProDH and EP-procyclins was determined by antibody detection in parasite homogenates. Briefly, parasites were harvested as described above and resuspended in lysis buffer that contained: 20 mM Tris-HCl pH 7.9, 1 mM EDTA pH 8.0, 0.25 M sucrose, 50 mM NaCl, 5% (v/v) glycerol, 1% (v/v) Triton X-100, 1 mM PMSF, 10 μg/ml aprotinin and 10 μg/ml leupeptin. Samples were chilled on ice (for 40 min) and clarified by centrifugation (15,000 *g* for 15 min at 4°C). Protein concentration was determined by the Bradford method using bovine serum albumin (BSA) as a standard [69]. Samples were submitted to protein electrophoresis (SDS-PAGE) and an equal amount of protein (30 μg) was loaded per lane. Proteins were transferred into 0.2 μm PVDF membranes (Amersham, GE, Life Sciences), blocked with PBS buffer plus 0.3% (v/v) Tween-20 (PBST) supplemented with 5% (w/v) skimmed milk powder and probed (16h at 4°C) against specific sera. The enzyme TbP5CDH was probed with a polyclonal specific serum (1:4,000) raised against its *T*. *cruzi* ortholog (TcP5CDH, TritrypDB accession number: Tc00.1047053510943.50) [28]. The enzyme P5C-synthase was probed with a polyclonal serum (1:3,000) produced in mouse against its close species *T*. *cruzi*, (TcP5CS, access code: TCSYLVIO_005298) exactly as previously described [28]. For digitonin assays, extracted fractions were probed with rabbit polyclonal antibodies against *T*. *brucei* ASCT (1:1,000), enolase (1:10,000), PPKD (1:1,000) and ProDH (1:500). EP-procyclins were probed with the monoclonal mAb-247 (1:1,500), which recognizes the EP-repeats of *T*. *brucei* procyclins (generous gift from Dr Terry W. Pearson, University of Victoria, Canada) [57]. As loading controls, two different polyclonal antisera were used: the mouse anti-TcGAPDH (1:3,000) and anti-HSP60 (access code: Tb427.10.6400) (1:2,000), dissolved in PBST-skim milk. Membranes were washed three times and incubated with goat anti-mouse IgG horseradish peroxidase (Sigma) diluted in PBST (1:50,000). Developing was done by using SuperSignal West Pico Chemiluminescent ECL substrate (Thermo Scientific) following the manufacturer’s instructions.

### Immunofluorescence microscopy

PCFs were cultured up to mid-exponential growth phase in SDM79. After this, parasites were washed with Voorheis’s modified PBS buffer (vPBS: 137 mM NaCl, 3 mM KCl, 16 mM Na_2_HPO_4_, 3 mM KH_2_PO_4_, 46 mM sucrose, 10 mM glucose) and harvested by centrifugation (850 *g* for 10 min at 4°C). Fixation, permeabilization and blocking were performed on poly-lysine coated glass slides, as previously described [71]. For antibody staining, polyclonal antisera produced against TbProDH (1:200) and TcP5CDH (1:250) [28] were dissolved in vPBS containing 20% (v/v) FBS and incubated for 2h at room temperature. Slides were washed five times with PBS and then incubated with AlexaFluor488-coupled goat anti-mouse IgG (Invitrogen) secondary antibody (1:600) plus TexasRed-X conjugated goat anti-mouse IgG (H+L) (Invitrogen) (1:400) for 1h. DNA staining was performed by adding 10 μg/ml of Hoechst probe (Invitrogen) and incubated for 5 min. Next, 2μl Fluoromount-G (GE, Healthcare) was added and a cover slip was mounted. Trypanosomes were visualized in a Leica DMi8 fluorescence microscope (Leica Microsystems) under an apochromatic 40x magnification lens. Image overlaying was done in imageJ software (NIH, Bethesda, MA, USA).

### Fluorescence Activated Cell Sorting (FACS) analysis

Wild type and ^*RNAi*^TbP5CDH (tet^-/+^) PCFs (5x10^6^ parasites) grown (3 days) in complete SDM79, as described above, were harvested (2,000*g* for 10 min at 4°C), washed twice with cold PBS, resuspended in 500 μl fixing solution [2% (v/v) formaldehyde and 0.05% (v/v) glutaraldehyde in PBS] and incubated for 20 min, as described before [72]. After fixation, parasites were washed twice and blocked in 200 μl PBS plus 2% (w/v) BSA (PBS-BSA) for 1h. Then, the cells were incubated with 200 μl monoclonal anti-EP procyclin solution (mAb 247 diluted 1:500 in PBS-BSA) for 2h [57]. After three washings with PBS, cells were incubated with a secondary antibody solution that contained goat anti-mouse IgG AlexaFluor-488 (Invitrogen) (1:1,000 in PBS-BSA) for 1h and were protected from light. Flow cytometry analysis was performed in a FACSCalibur flow cytometer (Becton Dickenson). FACS-acquired data were normalized using the unstained cells and only secondary antibodies provided as controls in the FlowJo v10 software (Tree Star, Inc.).

### Analysis of mitochondrial functions

To analyze the mitochondrial functions in wt and ^*RNAi*^TbP5CDH PCF cells, three parameters were taken into account: mitochondrial inner membrane potential (ΔΨ_m_), control of respiration and total ATP levels. After culture, cells were prepared as follow: parasites were harvested by centrifugation (1,000 *g* for 7 min at RT°) and dissolved in buffer A with glucose (BAG: 116 mM NaCl, 5.4 mM KCl, 0.8 mM MgSO_4_, 50 mM HEPES-KOH, pH 7.2 and 5.5 mM D-glucose), as previously described [30]. Final densities were adjusted to 10^9^ parasites/ml in BAG and kept on ice until further use. Parasite aliquots of 50 μl (5x10^7^ cells) of each group were used for measurements. The ΔΨ_m_ determinations was made spectrofluorometrically in parasites dissolved in cell respiration medium (CRM: 125 mM sucrose, 65 mM KCl, 10 mM HEPES-KOH pH 7.2, 1 mM MgCl_2_, 2.5 mM potassium phosphate) supplemented with 5 mM L-proline, 10 μM EGTA, 20% (w/v) non-fatty acids BSA (NFA-BSA) (Sigma) and 10 μM of the safranin-o dye (Sigma), as previously described [73]. Changes in the fluorescence were recorded on a Hitachi 2500 spectrofluorometer (λ_exi_496nm, λ_emi_586 nm) at 28°C under constant stirring. Oxygen consumption was determined using a high-resolution oxygraph (O2k, OROBOROS Instruments, Innsbruck, Austria), under constant stirring in a 2.1 ml final volume at 28°C. The reaction buffer was supplemented with NFA-BSA and EGTA as mentioned above. Assays were initiated by adding 5x10^7^ parasites to the oxygraph chamber. After adding the cells to the tightly closed oxygen-chamber, preparations were supplemented with 5 mM succinate or 5 mM proline, as indicated in each experiment. In order to measure parameters at mitochondrial levels, parasite suspensions were further permeabilized by adding 40 μM digitonin. Data were recorded using DatLab software (O2k, OROBOROS). In both measurements, additions of uncoupler or respiratory complex inhibitors were made as detailed in each experiment. ATP levels were determined using a luciferase bioluminescence assay (Sigma) according to the manufacturer’s indications. Briefly, the cells were harvested by centrifugation (2,000 *g* for 10 min at 4°C), washed twice with cold PBS and resuspended in the kit lysis buffer according to manufacturer´s instructions (Sigma). The intracellular ATP contents were extrapolated from a standard curve with known concentrations of ATP disodium salt. Results were obtained from four separate biological replicates (n = 3). Statistical analysis was performed using a one-way ANOVA test in GraphPad Prism v5.0a for Mac OS X (GraphPad Software, USA).

### Proton-Nuclear magnetic resonance analysis

PCFs use of glucose and proline as carbon sources was evaluated by nuclear magnetic resonance (proton-NMR) for the excreted end-products. Wt and ^*RNAi*^TbP5CDH (tet^-/+^) PCFs (10^6^ parasites/ml) were grown in complete SDM79 medium for 72h. Then, parasites were harvested by centrifugation (1,300 *g* for 10 min at 4°C) and washed twice with PBS. Then, 5x10^8^ parasites were transferred to 5 ml PBS supplemented or not with 4 mM L-proline + 4 mM D-[U-^13^C]-glucose. After 6h incubation at 26°C, cell suspensions were centrifuged and supernatants were submitted to NMR analysis, after adding 50 μl maleate (20 mM) as an internal reference to a 500 μl aliquot of the collected supernatant. ^1^H-NMR spectra were performed at 125.77 MHz on a Bruker DPX500 spectrometer equipped with a 5 mm broadband probe head. Measurements were recorded at 25°C with an ERETIC (Electronic REference To access In vivo Concentrations) method, which provides an electronically-synthesized reference signal. Acquisition conditions were as follows: 90° flip angle, 5000 Hz spectral width, 32 K memory size and 9.3 s total recycle time. Measurements were performed with 256 scans for a total time of almost 40 min. Before each experiment, the phase of the ERETIC peak was precisely adjusted. Protons linked to acetate carbon C2 generate by ^1^H-NMR five resonances, a single peak (unenriched acetate) flanked by two doublets ([^13^C]-acetate).

## Supporting Information

S1 File Oligonucleotides sequences used in this study. (DOCX)

S2 File Mix reaction for proline biosynthetic assay. (DOCX)

S1 Fig Assessment of mitochondrial function in PCFs using succinate. The capacity of the mitochondrial inner membrane to retain safranine dye was monitored in digitonized cells. Changes in the safranine fluorescence are representative of the mitochondrial inner membrane potential (ΔΨ_m_) in PCFs. ^*RNAi*^TbP5CDH tet^-/+^ cells were permeabilized with digitonin until fluorescence quenching was stabilized. Then, additions were made keeping the same concentrations detailed in the legend of Fig 4.
(EPS)

S2 Fig Kinetics of TbP5CDH in cell-free homogenates from PCFs. Enzyme activity rates for TbP5CDH as a function of P5C/γGS (A) and cofactor (NAD^+^) concentrations (B). Initial velocities were determined by varying the P5C/γGS concentration (20–600 μM) in the presence of 1 mM NAD^+^. NAD^+^ (20–2,500 μM) dependence was assayed in the presence of 600 μM P5C/γGS using potassium phosphate buffer, pH 7.2. The plot represents the mean ±SD of calculated velocities from three replicates. Values were adjusted to the Michaelis-Menten fitting using the Prism 5 for Mac OS X (GraphPad, Software, Inc.).
(TIF)

S3 Fig Tsetse midgut infection rates in wt parasites. Teneral flies were infected with a blood meal that contained 5x10^5^ wt PCFs/ml, containing or not tet (wt tet^-/+^ cells). Bars represent the percentage of trypanosome-infected flies as scored (S1-S4) by microscopy. The sum of each scored infection represents the total percentage of infected flies per treatment (total). The number of dissected flies (n) for each group were: wt tet^-^ n = 80, wt tet^+^ n = 75.
(TIFF)

S4 Fig Analysis procyclin expression. Positive-ion MALDI-TOF-MS analysis of procyclins after removal of the GPI anchors. 1-butanol extracts from wild-type (wt) (A-B) or TbP5CDH cells (D-E), grown in the absence (A and C) or the presence of tetracycline (tet) (B and D) were subjected to 48% aqueous hydrofluoric acid dephosphorylation followed by mild trifluoroacetic acid hydrolysis to remove the GPI anchors and generate EP procyclin peptides (63). The resulting polypeptides, corresponding to the C-terminal portions of procyclins, were analyzed by positive-ion MALDI-TOF-MS in a Shimadzu Axima TOF^2^, using sinapinic acid matrix. EP isoforms EP1-2 and EP3 are represented by respective pair of C-termini fragments containing (P(EP)_25_G-Etn) and (PDP(EP) _22_G-Etn) (63). Western blotting analysis of EP (E) and GPEET (F) expression of the same parasite butanol extracts used for MALDI-TOF analysis. Blottings were processed for chemiluminescent detection as described in the Materials Methods section, using anti-EP mAb-247 (1:1,250) and neat hybridoma supernatant for the 9G4 anti-GPPET mAb.
(TIFF)
