# Supplementary material for: Proline Metabolism is Essential for Trypanosoma brucei brucei Survival in the Tsetse Vector
Source: PLoS Pathog. 2017 Jan 23;13(1):e1006158. doi: 10.1371/journal.ppat.1006158 (PMC5289646; doi:10.1371/journal.ppat.1006158)
Supplement: S1 File — (DOCX) [file ppat.1006158.s001.docx]

S1.

Oligonucleotides sequences used in this study:

(*TbP5CDH*-sense) 5ʹ-GGTGTTTGAGGAGGCGGGGC-3ʹ

(*TbP5CDH*-antisense) 5ʹ-ACGCCAGCAAGCTCGGGATG-3ʹ

(*TbGAPDH*-sense) 5ʹ-CCGTGTTCCCACGGCTGATGT-3ʹ

(*TbGAPDH*-antisense) 5ʹ-TTGGAGGCGCGCTTCAGGG-3ʹ

(∆P5C-sense) 5ʹ-CTCGAGATGCTTCGCCGTACGTTGC-3ʹ

(∆P5C-antisense) 5ʹ-AAGCTTCACCGAAAATCGAAGGAAGTCGC-3ʹ
