# Supplementary material for: Proline Metabolism is Essential for Trypanosoma brucei brucei Survival in the Tsetse Vector
Source: PLoS Pathog. 2017 Jan 23;13(1):e1006158. doi: 10.1371/journal.ppat.1006158 (PMC5289646; doi:10.1371/journal.ppat.1006158)
Supplement: S2 File — (DOCX) [file ppat.1006158.s002.docx]

S2. Mix reaction for proline biosynthetic assay

Final volumes were adjusted to 1 ml with PBS and prepared as follows:

- (L-Glu) 20 mM sodium glutamate, 10 mM MgCl_2_, 0.5 mM NADPH, 5 mM phospho(enol)pyruvic acid cyclohexylammonium (PEP) as an ATP-regeneration system;
- (DL-P5C/γGS) 1.5 mM DL-P5C (freshly prepared), 0.5 mM NADPH;
- (L-Gln) 5 mM L-glutamine, 10 mM MgCl_2_, 0.5 mM NADPH, 5 mM phospho(enol)pyruvic acid cyclohexylammonium (PEP) as an ATP-regeneration system;
- (L-Ala) 5 mM L-alanine, 10 mM MgCl_2_, 0.5 mM NADPH and 5 mM PEP;
- (L-Arg) 5 mM L-arginine, 1 mM MnCl_2_ and 50 µM pyridoxal.
