# Supplementary figures and images for: Proline Metabolism is Essential for Trypanosoma brucei brucei Survival in the Tsetse Vector
Source: PLoS Pathog. 2017 Jan 23;13(1):e1006158. doi: 10.1371/journal.ppat.1006158 (PMC5289646; doi:10.1371/journal.ppat.1006158)

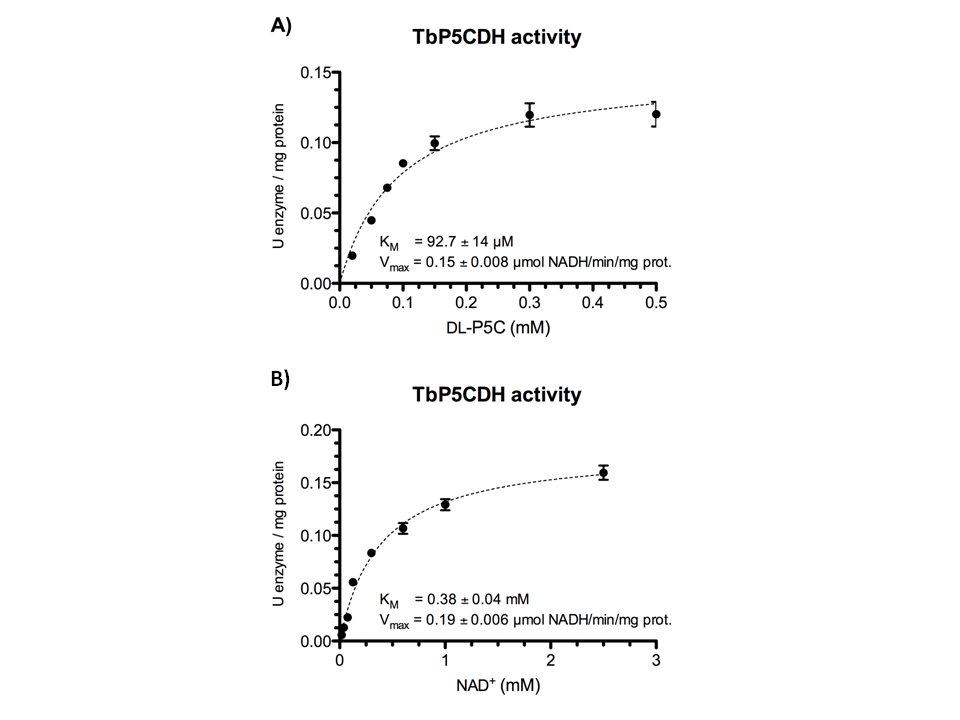

Supplement: S2 Fig — Enzyme activity rates for TbP5CDH as a function of P5C/γGS (A) and cofactor (NAD+) concentrations (B). Initial velocities were determined by varying the P5C/γGS concentration (20–600 μM) in the presence of 1 mM NAD+. NAD+ (20–2,500 μM) dependence was assayed in the presence of 600 μM P5C/γGS using potassium phosphate buffer, pH 7.2. The plot represents the mean ±SD of calculated velocities from three replicates. Values were adjusted to the Michaelis-Menten fitting using the Prism 5 for Mac OS X (GraphPad, Software, Inc.). (TIF) [file ppat.1006158.s004.tif]

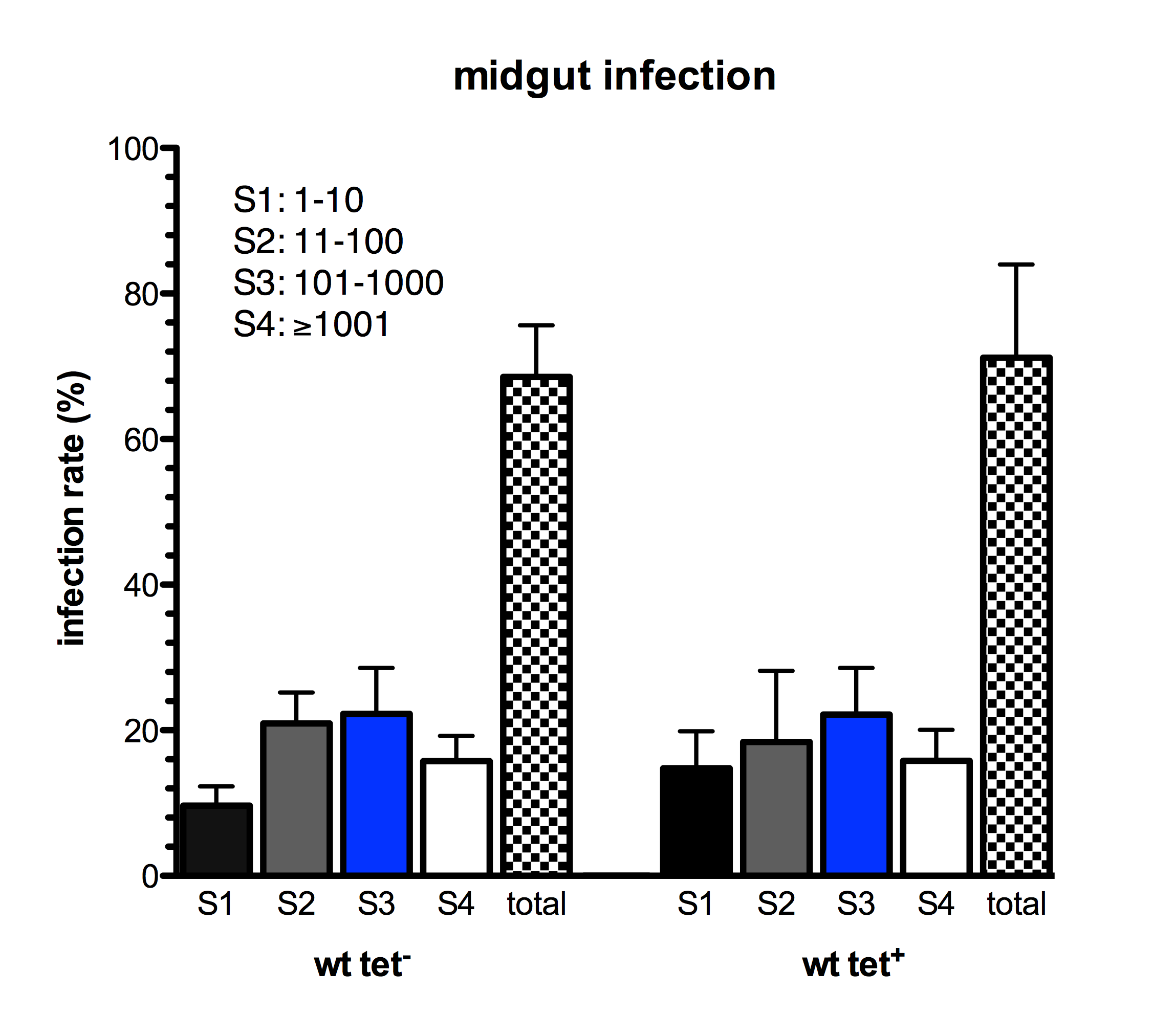

Supplement: S3 Fig — Teneral flies were infected with a blood meal that contained 5x10^5^ wt PCFs/ml, containing or not tet (wt tet-/+ cells). Bars represent the percentage of trypanosome-infected flies as scored (S1-S4) by microscopy. The sum of each scored infection represents the total percentage of infected flies per treatment (total). The number of dissected flies (n) for each group were: wt tet- n = 80, wt tet+ n = 75. (TIFF) [file ppat.1006158.s005.tiff]

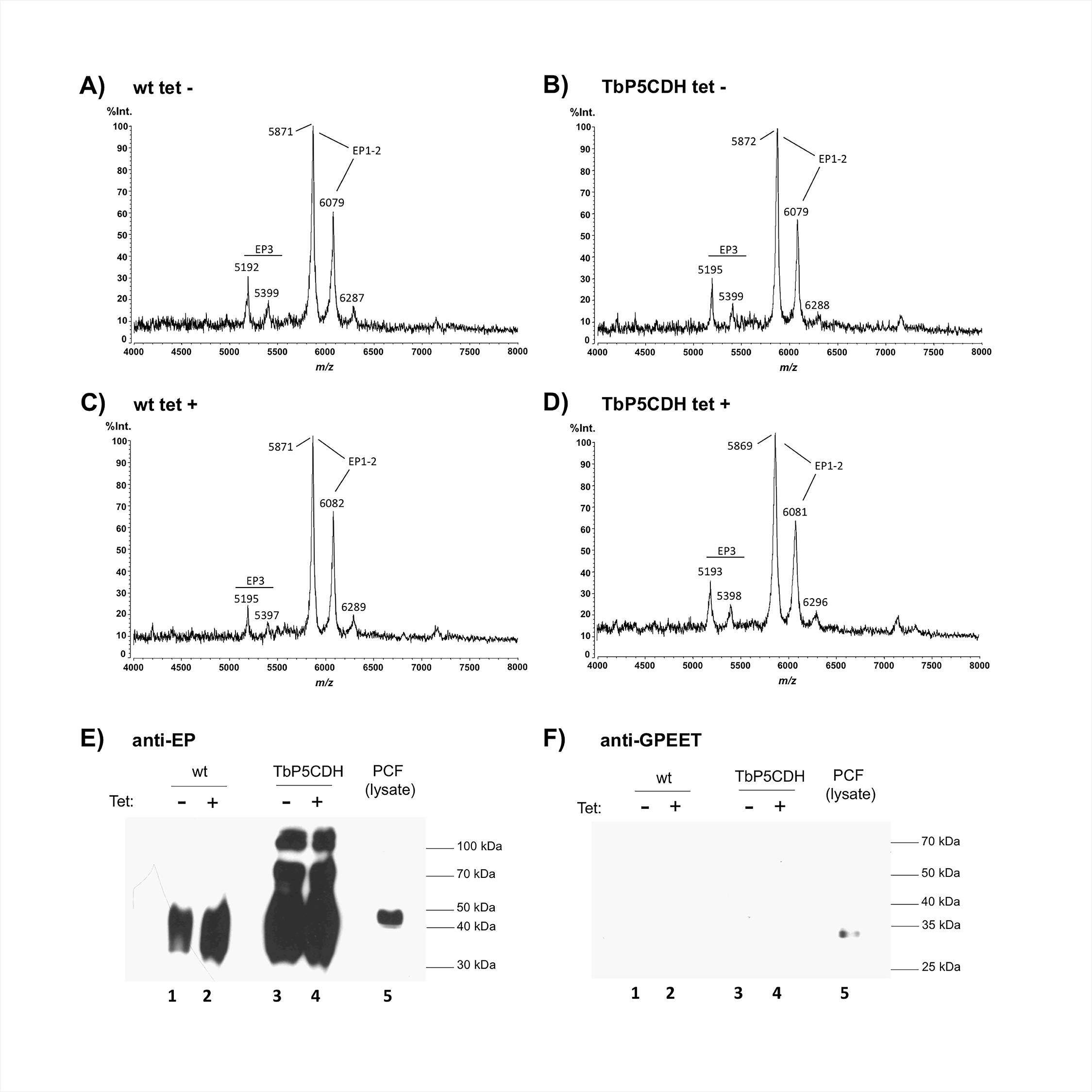

Supplement: S4 Fig — 1-butanol extracts from wild-type (wt) (A-B) or TbP5CDH cells (D-E), grown in the absence (A and C) or the presence of tetracycline (tet) (B and D) were subjected to 48% aqueous hydrofluoric acid dephosphorylation followed by mild trifluoroacetic acid hydrolysis to remove the GPI anchors and generate EP procyclin peptides (63). The resulting polypeptides, corresponding to the C-terminal portions of procyclins, were analyzed by positive-ion MALDI-TOF-MS in a Shimadzu Axima TOF2, using sinapinic acid matrix. EP isoforms EP1-2 and EP3 are represented by respective pair of C-termini fragments containing (P(EP)25G-Etn) and (PDP(EP) 22G-Etn) (63). Western blotting analysis of EP (E) and GPEET (F) expression of the same parasite butanol extracts used for MALDI-TOF analysis. Blottings were processed for chemiluminescent detection as described in the Materials Methods section, using anti-EP mAb-247 (1:1,250) and neat hybridoma supernatant for the 9G4 anti-GPPET mAb. (TIFF) [file ppat.1006158.s006.tiff]
